# Isolation, whole-genome sequencing, and annotation of two antibiotic-producing and antibiotic-resistant bacteria, *Pantoea rodasii* RIT 836 and *Pseudomonas endophytica* RIT 838, collected from the environment

**DOI:** 10.1371/journal.pone.0293943

**Published:** 2024-02-27

**Authors:** Serena Tuytschaevers, Leila Aden, Zacchaeus Greene, Chanei Nixon, Wade Shaw, Dillan Hatch, Girish Kumar, Renata Rezende Miranda, André O. Hudson

**Affiliations:** 1 Thomas H. Gosnell School of Life Sciences, Rochester Institute of Technology, Rochester, New York, United States of America; 2 Rochester Prep High School, Rochester, New York, United States of America; 3 School of Chemistry and Materials Science, Rochester Institute of Technology, Rochester, New York, United States of America; Tianjin University, CHINA

## Abstract

Antimicrobial resistance (AMR) is a global threat to human health since infections caused by antimicrobial-resistant bacteria are life-threatening conditions with minimal treatment options. Bacteria become resistant when they develop the ability to overcome the compounds that are meant to kill them, i.e., antibiotics. The increasing number of resistant pathogens worldwide is contrasted by the slow progress in the discovery and production of new antibiotics. About 700,000 global deaths per year are estimated as a result of drug-resistant infections, which could escalate to nearly 10 million by 2050 if we fail to address the AMR challenge. In this study, we collected and isolated bacteria from the environment to screen for antibiotic resistance. We identified several bacteria that showed resistance to multiple clinically relevant antibiotics when tested in antibiotic susceptibility disk assays. We also found that two strains, identified as *Pantoea rodasii* RIT 836 and *Pseudomonas endophytica* RIT 838 via whole genome sequencing and annotation, produce bactericidal compounds against both Gram-positive and Gram-negative bacteria in disc-diffusion inhibitory assays. We mined the two strains’ whole-genome sequences to gain more information and insights into the antibiotic resistance and production by these bacteria. Subsequently, we aim to isolate, identify, and further characterize the novel antibiotic compounds detected in our assays and bioinformatics analysis.

## Introduction

The United States (US) Centers for Disease Control and Prevention (CDC) has reported that antimicrobial resistance (AMR) is a threat to public health worldwide [1, 2]. More than 2.8 million antimicrobial-resistant infections occur each year in the US alone [1]. It is estimated that, in 2019, there were 1.27 million deaths caused by resistant infections, and, by 2050, this number could increase to 10 million deaths annually if no global action is taken against AMR [3, 4].

Antibiotics are medicines that are designed to prevent and treat bacterial infections by making it difficult for bacteria to grow and divide [2, 5–7]. Specifically, antimicrobials can interfere with cell wall synthesis, inhibit protein synthesis, interfere with nucleic acid synthesis, or inhibit a metabolic pathway [8, 9]. For example, β-lactams (such as penicillin) and other antibiotics (such as vancomycin) act via the first mechanism by inhibiting the cross-linking of amino acid chains in peptidoglycans, thus compromising cell wall synthesis [10, 11]. Aminoglycosides, tetracycline, chloramphenicol, among others, inhibit protein biosynthesis by targeting one of the subunits of the bacterial ribosome [12]. Additionally, sulfonamides/trimethoprim and quinolones prevent nucleic acid synthesis by inhibiting essential enzymes for folic acid production and DNA replication, respectively [13].

AMR can impact anyone because pathogens can spread between people, animals, and the environment [14, 15]. AMR happens when pathogens develop the capacity to overcome the drugs that were meant to kill them [1, 16]. Some ways by which bacteria can overcome the effects of antimicrobial agents are: (1) enzymatic inactivation of antibiotics, such as β-lactamase production; (2) efflux mechanisms, where these compounds are exported from the bacterial cell into the external environment; (3) decreased antibiotic uptake by alteration of the cellular permeability; (4) target site modification; and (5) biofilm production [17, 18]. Resistance can be either intrinsic, mutation associated, or passed from one organism to another via various genetic mechanisms [19]. Bacteria can exchange resistance genes horizontally (conjugation), intake DNA from the environment (transformation), or acquire genetic material upon infection by a virus or viral vector (transduction) [20, 21]. With the spread of AMR, some bacteria, also called “superbugs”, become multi- or pan-resistant to antibiotics, and there are very limited options left to treat infections caused by these organisms [5, 22].

A significant increase in multi-drug resistant (MDR) pathogens worldwide cannot be counteracted by the decreasing progress in the development of new antimicrobial agents [23–25]. In the early 20^th^ century, one of the most common causes of death was illnesses caused by infectious agents [23]. Antibiotic development also started around this time, with many of the drugs in use nowadays being discovered during the 1940s and 1960s, a period also known as the "Golden Age” of antibiotic discovery [23, 24, 26–28]. After 1962, when nalidixic acid was introduced, there has been an innovation gap or discovery void of over 40 years, when only two major new classes of antibiotics have been commercialized [29, 30]. Only about 10% of drug candidates currently in clinical trials are new antibacterial compounds. Among these, less than 25% represent a novel class or work via a new mechanism of action [31]. Therefore, new strategies and investment in antibiotic discovery and development are urgently needed to tackle AMR.

Governmental agencies in the US provide funding to help state and local health departments to detect and prevent AMR threats, as well as invest money in institutions for innovations, therapeutics, and diagnosis [32]. According to the 2019 Antimicrobial Resistance Threats Report, prevention and control efforts in the US reduced deaths from antimicrobial-resistant infections by 18% [2, 33]. Overall, there had been significant progress in the fight against AMR until the emergence of the SARS-CoV-2 (COVID-19) pandemic. A 2022 CDC report showed the dramatic impact that the COVID-19 pandemic had on the increase of AMR in the US, stating that a lot of progress was lost [33]. The high incidence of secondary infections, often caused by MDR bacteria, coupled to the substantial increase in antibiotic use (mostly unnecessarily) as treatment adjuvants, likely contributed to this scenario and intensified the urgency of new therapeutic agents and strategies to fight MDR bacteria and stop AMR spread [4, 9, 21, 24, 31, 34].

Another potential source of MDR bacteria and, consequently, cause of AMR spread is the environment. The increased use of antibiotics both in the clinic and agriculture plays an important role in the spread of AMR genes among bacteria. When antibiotics are released into the environment through water and soil, they promote the selection and outgrowth of bacteria carrying antibiotic-resistant genes. These genes can be transferred to other bacteria in the environment, including disease-causing species. This process leads to substantial alterations in the antibiotic susceptibility of entire microbial communities and poses a significant threat to both human and animal health [35, 36]. Our goal with the present study was to screen the environment for the presence of bacteria that are resistant to clinically relevant antibiotics. We collected environmental samples around the Rochester Institute of Technology’s (RIT) campus (Rochester, NY, US) from diverse sources. From these samples, we identified two bacterial strains, *Pantoea rodasii* RIT 836 and *Pseudomonas endophytica* RIT 838, that were resistant to various commonly used antibiotics. Interestingly, we also show that these strains were capable to produce bactericidal activity against both Gram-positive and Gram-negative bacteria. Finally, we discuss the bioinformatics analysis of their microbial genomes using the antibiotics & Secondary Metabolite Analysis Shell (antiSMASH) tool to identify biosynthetic gene clusters (BGCs) and corresponding products that might be involved in the antimicrobial activities displayed by these bacteria [37, 38].

## Materials and methods

### Sample collection, processing, and bacterial growth and isolation

Samples were collected from various places within the RIT campus, such as tree moss, mulch, water fountain, and soil, in sterile 50 mL centrifuge tubes while wearing gloves. A small amount of each sample (the tip of a spatula) was incubated in Luria Broth (LB) overnight, shaking at 30°C, 100 rpm, under aerobic conditions. Serial dilutions (10^−1^ to 10^−10^) of each grown sample were prepared in liquid LB medium and 100 μL of each were plated onto LB agar plates and incubated overnight at 30°C.

### Antibiotic resistance screening

Four distinct isolated colonies were chosen from each sample to evaluate their antibiotic resistance profiles. Individual colonies were grown overnight in 5 mL liquid LB shaking at 30°C, 150 rpm. On the next day, each culture was pelleted at 6,000 rpm, room temperature, for 20 minutes, and the cell pellets were resuspended in 5 mL of sterile phosphate buffered saline (PBS), pH 7.4, to obtain an inoculum with OD_600_ of 0.1. The PBS bacterial suspensions were then used in the antibiotic disk susceptibility assays.

All bacterial strains were tested against seven commercially available antibiotics (Oxoid, UK): polymyxin B, 300 IU; sulfamethoxazole/trimethoprim, 25 mcg; chloramphenicol, 30 mcg; rifampicin, 5 mcg; clindamycin, 2 mcg; colistin sulfate, 10 mcg; and vancomycin, 30 mcg. Tetracycline (20 μL of a 10 mg/mL solution) and methanol (20 μL) were applied to blank paper discs (6 mm, BBL^™^) and used as positive and negative controls, respectively. For the assay, 40 mL of warm LB agar were inoculated with 400 μL of each bacterial suspension in PBS. Discs were then placed on the solidified agar and all plates were incubated at room temperature overnight. Plates were imaged in Chemidoc MP (Bio-Rad) using the colorimetric settings. The zone of inhibition (ZOI, mm) around each disc was measured using ImageJ (NIH). These assays were performed in duplicates and were analyzed using Microsoft Excel (Microsoft). Bacteria that showed resistance (ZOI = 0 mm) to four or more antibiotics were selected for further studies.

### Whole-genome extraction, sequencing, and annotation

Genomic DNA (gDNA) was isolated from fresh bacterial cultures in LB medium using the GenElute bacterial genomic DNA isolation kit (Sigma-Aldrich, USA) according to the manufacturer’s protocol. For sequencing, the gDNA was quantified using a NanoDrop spectrophotometer.

The library preparation for Illumina sequencing was performed using the Nextera XT library preparation kit (Illumina Inc., USA) following the manufacturer’s instructions. The average library insert size (in bps) was determined using an Agilent high sensitivity DNA chip on a 2100 Bioanalyzer instrument (Agilent Technologies, Santa Clara, USA). The library was quantified with a Qubit^®^ 3.0 fluorometer and diluted down to 16 pM. Sequencing was performed on an Illumina MiSeq platform (Illumina, San Diego, CA, USA) using V3 Kit for 2×300 cycles in the Genomics lab at RIT.

The quality control and preprocessing of the FASTQ files were performed using fastp. We removed the reads with quality score < 30. Filtered reads were assembled using Unicycler v0.5.0 which uses SPAdes v3.15.4 to assemble the short reads [39, 40]. The quality assessment of de novo genome assembly was evaluated by QUAST- a quality assessment tool [41]. Genome annotation of assemblies was performed using the PGAP (Prokaryotic Genome Annotation Pipeline) which is integrated into the NCBI RAPT [Read assembly and Annotation Pipeline Tool; https://www.ncbi.nlm.nih.gov/rapt (accessed on 06 December 2022)].

### Scanning Electron Microscopy (SEM)

Samples for SEM analysis were prepared following a previously reported procedure [42]. 10 μL of overnight cultures of the bacteria in LB were used for each sample. The cells were soaked in a fixative solution (2% glutaraldehyde in phosphate buffered saline (PBS) pH 7.4) for 45 min at room temperature, then washed 3 × 5 min using the same solution. Next, the samples were dehydrated in 50–80% graded ethanol for 10 min each, followed by 2 × 5 min washes with 95% ethanol, and 3 × 15 min washes with fresh 100% ethanol. All liquid was removed by pipetting and the samples were stored (sealed with Parafilm) at room temperature overnight. Prior to SEM, the samples were coated for 2 min with gold-palladium using an SPI sputter coater to mitigate charging in the electron beam. SEM was performed at a voltage of 5 kV using a Mira3 Tescan field-emission SEM from the Nanoimaging Lab at the Rochester Institute of Technology.

### Preparation of bacterial organic extracts for antibiotic activity testing

Selected bacterial strains were grown in starter cultures of 5 mL LB overnight, shaking at 30°C, 150 rpm. The liquid cultures were scaled-up to 100 mL LB, grown under the same conditions, followed by 1 L of LB, growing for 48 h, shaking at 30°C, 150 rpm. Each 1 L LB liquid culture was pelleted at 6,000 rpm for 20 minutes at 4°C, and the supernatant was decanted from the cell pellet. The supernatant was acidified to pH <2 and sodium chloride was added until saturation. Extractions were performed with 250 mL of ethyl acetate per 1 L of media, and the organic layers were dried with anhydrous sodium sulfate. After filtration, the dry organic layers were concentrated to crude residues using a rotary evaporator (BUCHI), resuspended in methanol and dried using a Speed-vac (Eppendorf). Blank extractions were also performed using 3 L of uninoculated LB medium to serve as controls in the assays.

### Antibiotic activity of bacterial organic extracts against *Escherichia coli*

Reference strains (*Escherichia coli* ATCC 25922, *Staphylococcus aureus* ATCC 25923, *Pseudomonas aeruginosa* ATCC 27853, and *Bacillus subtilis* BGSC 168) were grown in 5 mL liquid LB shaking at 37°C, 150 rpm, for 16 h. The cultures were pelleted at 6,000 rpm, room temperature, for 20 minutes, and the cell pellets were resuspended in 5 mL of sterile PBS, pH 7.4, to obtain an inoculum with OD_600_ of 0.1. The PBS bacterial suspensions were then used in the disk assays. A volume of 400 μL of each culture’s PBS suspension was added to 40 mL of warm LB agar. Six blank paper discs (6 mm, BBL^™^) were placed on the agar and different solutions were pipetted onto the discs, as follows: 5, 10, and 20 μL of bacterial extracts (250 mg/mL in methanol); 20 μL of blank LB extracts (250 mg/mL in methanol); 20 μL of tetracycline (10 mg/mL; positive control); and 20 μL of methanol (negative control). Plates were incubated at 37°C for 16 h, imaged in Chemidoc MP (Bio-Rad) using the colorimetric settings, and the ZOI values were measured using ImageJ (NIH). These assays were performed in duplicates and were analyzed using Microsoft Excel (Microsoft).

### Predictions of secondary metabolite production of bacterial strains via bioinformatics analyses

#### RIT 836 and RIT 838

The FASTA files corresponding to each bacterium’s whole-genome sequences were uploaded to antiSMASH (version 6.1.1) with all extra features enabled to detect potential gene clusters involved in secondary metabolite biosynthesis. The strictness criterion was set at relaxed to allow for the discovery of clusters encoding less characterized metabolites [37, 38].

#### Control strains

Three strains of *Pantoea rodasii* (NCBI accession number: PIQI00000000, MLFP00000000, JTJJ00000000) and one strain of *Pseudomonas endophytica* (NCBI accession number: LLWH00000000) were uploaded to antiSMASH (7.0.1) by using their NCBI accession number to get each desired sequence. All extra features were enabled to detect potential gene clusters involved in secondary metabolite biosynthesis. The strictness criterion was set at relaxed to allow for the discovery of clusters encoding less characterized metabolites [37, 38].

## Results and discussion

### Sample collection, processing, and bacterial growth and isolation

All environmental samples except for the water showed bacterial growth, which was expected since a water fountain is in constant movement, making it not an ideal place for bacteria to grow and proliferate. After serial dilutions and plating of the bacteria from the remaining three samples, all plates showed growth, with individual colonies being observed in the highest serial dilutions (10^−8^, 10^−9^, 10^−10^). It is important to note that we only cultivated the bacteria under aerobic conditions. We therefore acknowledge that this potentially limited the number and diversity of unique bacteria detected in our samples, excluding, for instance, anaerobic species that could have been found in the screened environment, particularly in the soil [43].

### Antibiotic resistance screening

**Fig 1:**
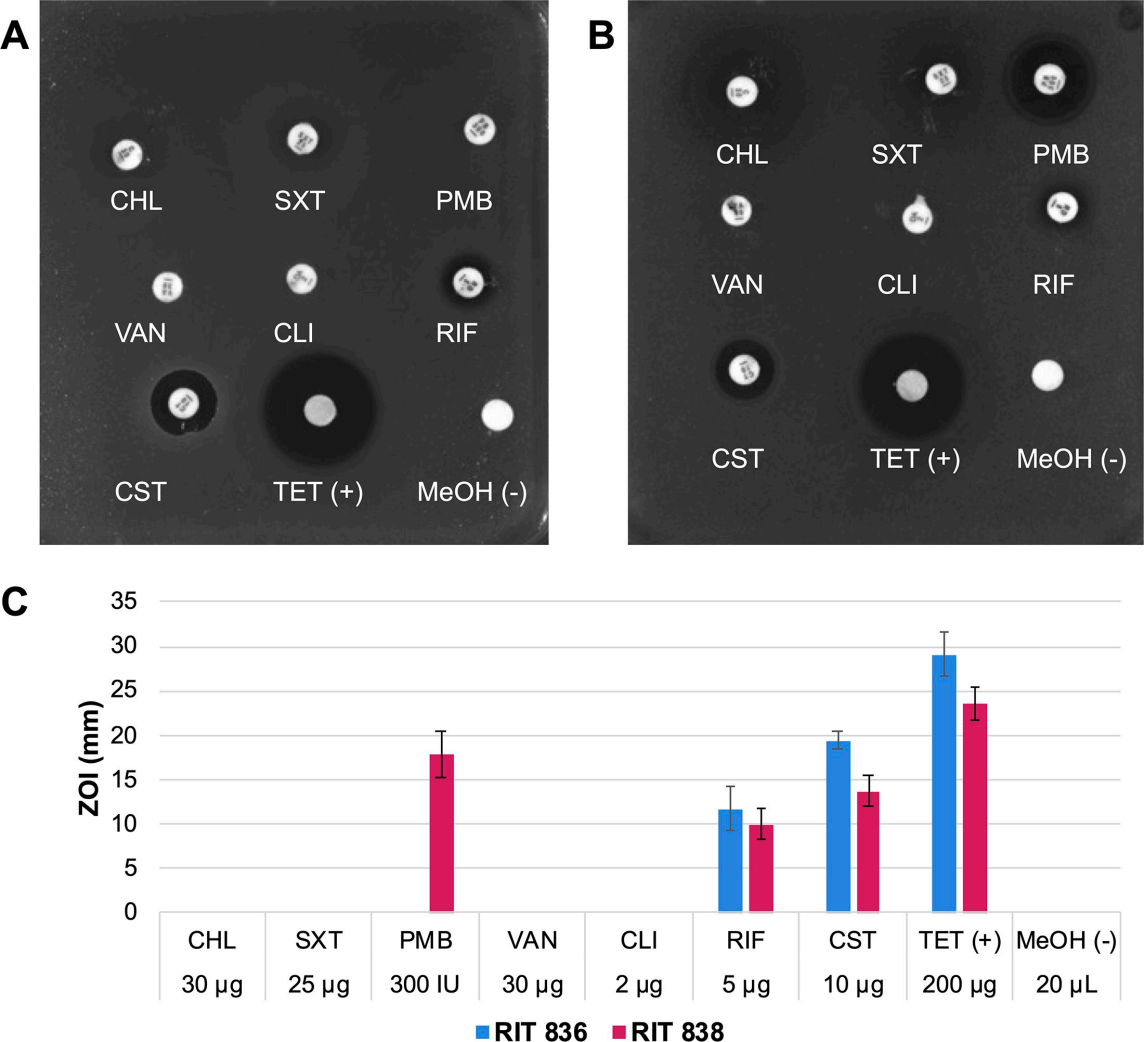
Disc-diffusion susceptibility assays of RIT 836 (**A**) and RIT 838 (**B**), each treated with chloramphenicol, 30 μg (CHL); sulfamethoxazole/trimethoprim, 25 μg (SXT); polymyxin B, 300 IU (PMB); vancomycin, 30 μg (VAN); clindamycin, 2 μg (CLI); rifampicin, 5 μg (RIF); colistin sulfate, 10 μg (CST); tetracycline, 200 μg (TET, +); and methanol, 20 μL (MeOH, -). These experiments were performed in duplicates and the average ZOI values are shown in S1 Table. (**C**) Bar graph comparing the antibiotic susceptibility of RIT 836 and RIT 838 as shown in panels **A** and **B**.

We selected four distinct colonies from the two lowest serial dilution plates of each environmental sample. Our selection was based on the colony morphology, i.e., characteristics such as shape, size, color, and texture. Subsequently, we grew each individual colony in a Petri dish to ensure that each sample indeed produced uniform colonies before moving forward with our experiments. Each selected colony from each of the three environmental samples mentioned above (12 samples in total) to screen for antibiotic resistance in disc susceptibility assays using seven clinically relevant antibiotics, including chloramphenicol (CHL), sulfamethoxazole/trimethoprim (SXT), polymyxin B (PMB), vancomycin (VAN), clindamycin (CLI), rifampicin (RIF), and colistin sulfate (CST). One of the chosen colonies did not show any growth and was therefore excluded from the screening. The remaining 11 strains showed resistance to at least two antibiotics when examining the bacterial growth inhibition halos around the discs (S1 Fig, S1 Table). Interestingly, 9 of the 11 strains showed resistance to VAN and CLI (ZOI = 0 mm). Two bacteria (strains 3 and 8, named RIT 836 and RIT 838 from now on) showed resistance to at least four antibiotics and were selected to highlight here and utilize in further studies. RIT 836 was resistant to five antibiotics, including CHL, SXT, PMB, VAN, and CLI (Fig 1A and 1C, S1 Table). RIT 838 was resistant to four antibiotics, including CHL, SXT, VAN, and CLI (Fig 1B and 1C, S1 Table).

### Identification and characterization of bacterial strains

We performed whole-genome sequencing and annotation of the two selected strains, initially referred to RIT 836 and RIT 838, to identify their bacterial species. Illumina MiSeq yielded approximately 2.7 million reads for the studied sample. The genome coverage was 142 ×, which is sufficient to derive high-quality draft genome assemblies. Genome annotation and assembly statistics are presented in Table 1.

**Table 1:** Bacterial genome sequencing, assembly, and annotation results for *Pantoea rodasii* RIT 836 and *Pseudomonas endophytica* RIT 838.

Characteristic	*Pantoea rodasii* RIT 836	*Pseudomonas endophytica* RIT 838
GenBank accession no.	JAPVEE000000000	JARJDJ000000000
SRA accession	SRR22685145	SRR22685143
Assembly size (bp)	5,250,231	4,749,695
Coverage (**×**)	142	25
No. of contigs	29	52
*N*_50_ (bp)	582,425	164,368
Assembly GC content (%)	54.66	55.66
No. of genes	4,884	4,769
No. of tRNAs	68	72
No. of rRNAs	7	12
%gANI*	95.81	95.41

*gANI, genome-wide average nucleotide identity. All statistics are based on contigs of size ≥ 500 bp.

RIT 836, isolated from tree moss, was identified as *Pantoea rodasii*. RIT 838, isolated from mulch, was identified as *Pseudomonas endophytica*. The *Pantoea* genus comprises various bacterial species with many interesting capabilities, such as biodegradation, biosynthesis, and antibiotic production, which can be used for agricultural, environmental, and clinical applications [44]. The *Pseudomonas* genus is composed of gram-negative bacteria. *Pseudomonas* species are pathogenic to both animals and plants, such as *P*. *aeruginosa* and *P*. *tolaasii*, respectively [45, 46]. In fact, infections by *P*. *aeruginosa* can be life-threatening as this species often exhibits resistance to multiple drugs and virulence, being classified as one of the ESKAPE pathogens (*Enterococcus faecium*, *Staphylococcus aureus*, *Klebsiella pneumoniae*, *Acinetobacter baumannii*, *Pseudomonas aeruginosa*, and *Enterobacter* species) [47].

**Fig 2:**
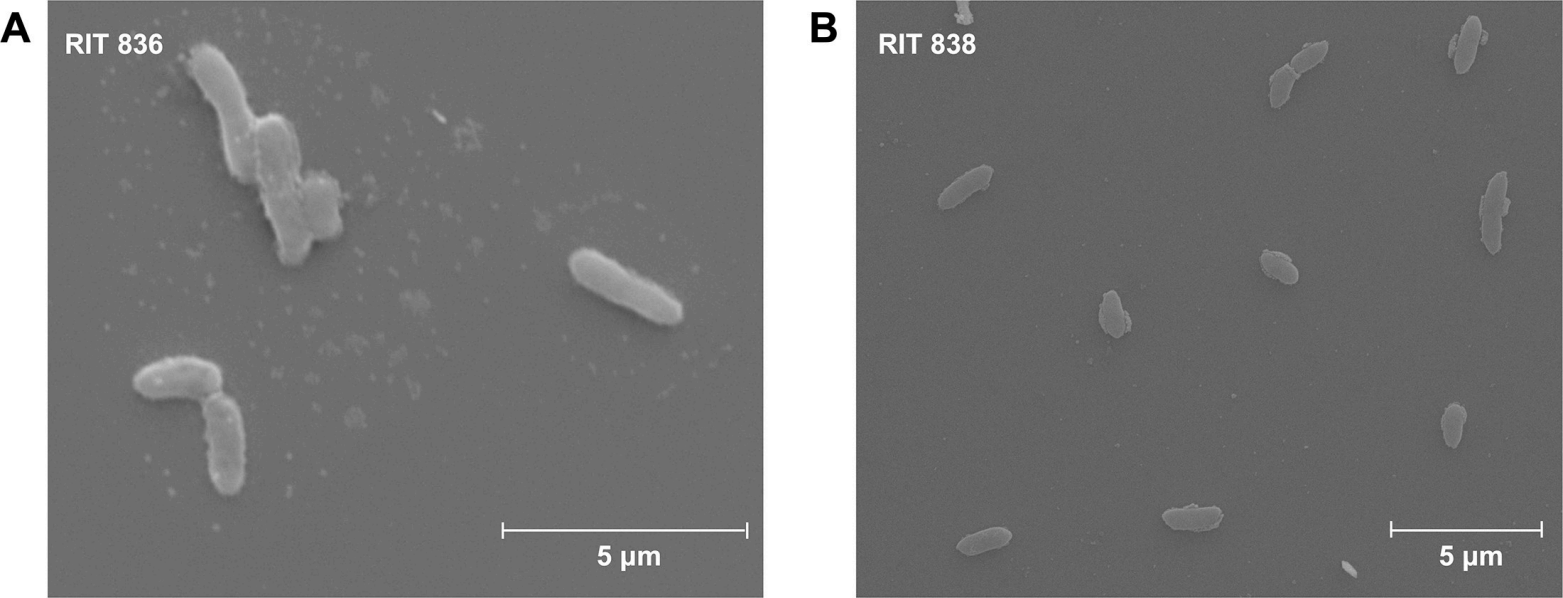
Scanning electron micrographs (SEM) showing *Pantoea rodasii* RIT 836 (×12,100 magnification) (**A**) and *Pseudomonas endophytica* RIT 838 (×12,000 magnification) (**B**) cells. Both bacteria are rod-shaped and of approximately 2.0 μm in diameter.

Additionally, we observed that, on LB agar, *P*. *rodasii* RIT 836 form yellow-orange colonies and *P*. *endophytica* RIT 838 form white colonies. Upon electron microscopy examination, *P*. *rodasii* RIT 836 show both individual and clumps of rod-shaped cells, about 2 μm in diameter (Fig 2A), and *P*. *endophytica* RIT 838 show individual rod-shaped cells that also measure approximately 2 μm in diameter (Fig 2B).

### Antibiotic activity screening of RIT 836 and RIT 838 organic extracts

**Fig 3:**
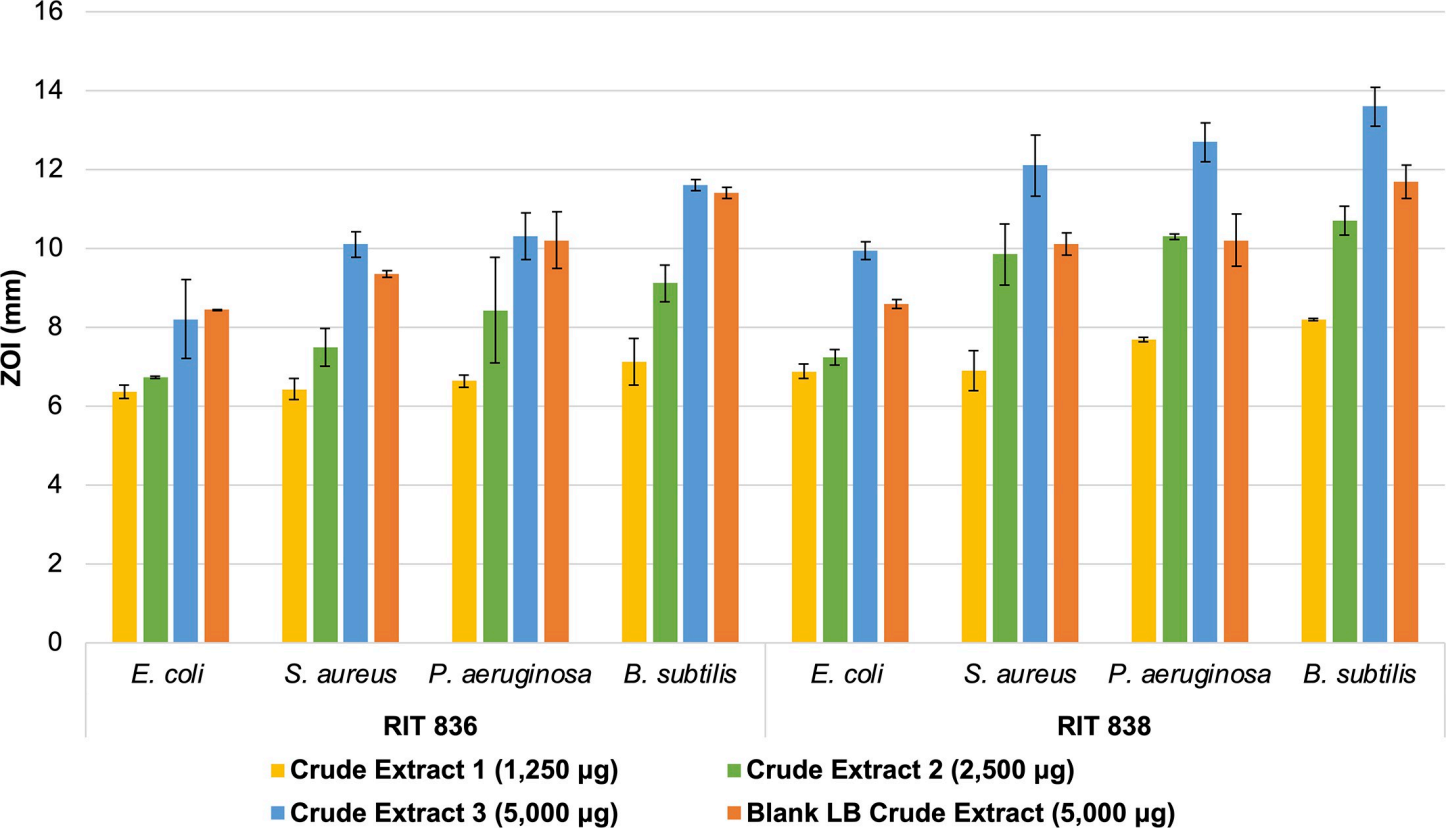
Comparison of the growth inhibitory activity (ZOI) of *P*. *rodasii* RIT 836 and *P*. *endophytica* RIT 838 against four bacteria, including both Gram-positive and Gram-negative species. The four bacteria were treated with increasing amounts of ethyl acetate spent medium crude extracts of RIT 836 and RIT 838 in disc-diffusion inhibitory assays. Blank LB (no bacteria) extract crude was used as a medium control. The data represent the mean values ± SD of two independent experiments and the error bars indicate the SD values. More details, including additional controls, are shown in the S2 and S3 Figs, S2 Table.

Ethyl acetate spent LB medium extracts of the two bacteria were tested for antimicrobial activity in disc-diffusion inhibitory assays against four reference strains, including Gram-positive (*Bacillus subtilis* and *Staphylococcus aureus*) and Gram-negative (*Escherichia coli* and *Pseudomonas aeruginosa*) microorganisms (S2 and S3 Figs). Increasing amounts of the crude extracts were applied to sterile discs equally spread-out on an agar plate inoculated with a reference strain and the ZOI (mm) around each disc was measured (S2 Table). The ZOI values increased with the amount of extract of both *P*. *rodasii* RIT 836 and *P*. *endophytica* RIT 838 (S2 and S3 Figs, S2 Table). When comparing the bacterial extracts to the medium control at the same concentration (Fig 3, Crude Extract 3 and Blank LB Crude Extract), the results suggest that RIT 838 caused a higher inhibitory activity than RIT 836 against all four tested strains. Furthermore, RIT 838’s showed bactericidal activity against both Gram-negative and Gram-positive species, indicating a broad-spectrum antimicrobial activity. It is worth mentioning that, due to our small sample number in these assays (duplicates), we were unable to perform a statistical analysis to confirm the significance of RIT 838 inhibitory activity against the tested strains.

### Predictions of secondary metabolite production by RIT 836 and RIT 838 genome mining

We used the bioinformatics/genomics tool known as antibiotics & Secondary Metabolite Analysis Shell (antiSMASH, 6.1.1) to identify secondary metabolite biosynthetic gene clusters (BGCs) present in the bacteria’s genomes that encode for potentially novel antibiotics [37, 38, 48]. BGCs are a clustered group of two or more genes in a genome that encode for a biosynthetic pathway for the production of a secondary metabolite [49, 50]. Different classes of BGCs include: redox-cofactors, aryl polyenes, hserlactones, thiopeptides, terpenes, type III PKS (T3PKS), ribosomally synthesized and post-translationally modified peptide product (RiPP-like), *N*-acetylglutaminylglutamine amid (NAGGN), tRNA-dependent cyclodipeptide synthases (CDPS), PPY-like pyrone (PpyS-KS), ranthipeptides, and nonribosomal peptide-synthetase-like (NRPS-like) [38, 49].

**Fig 4:**
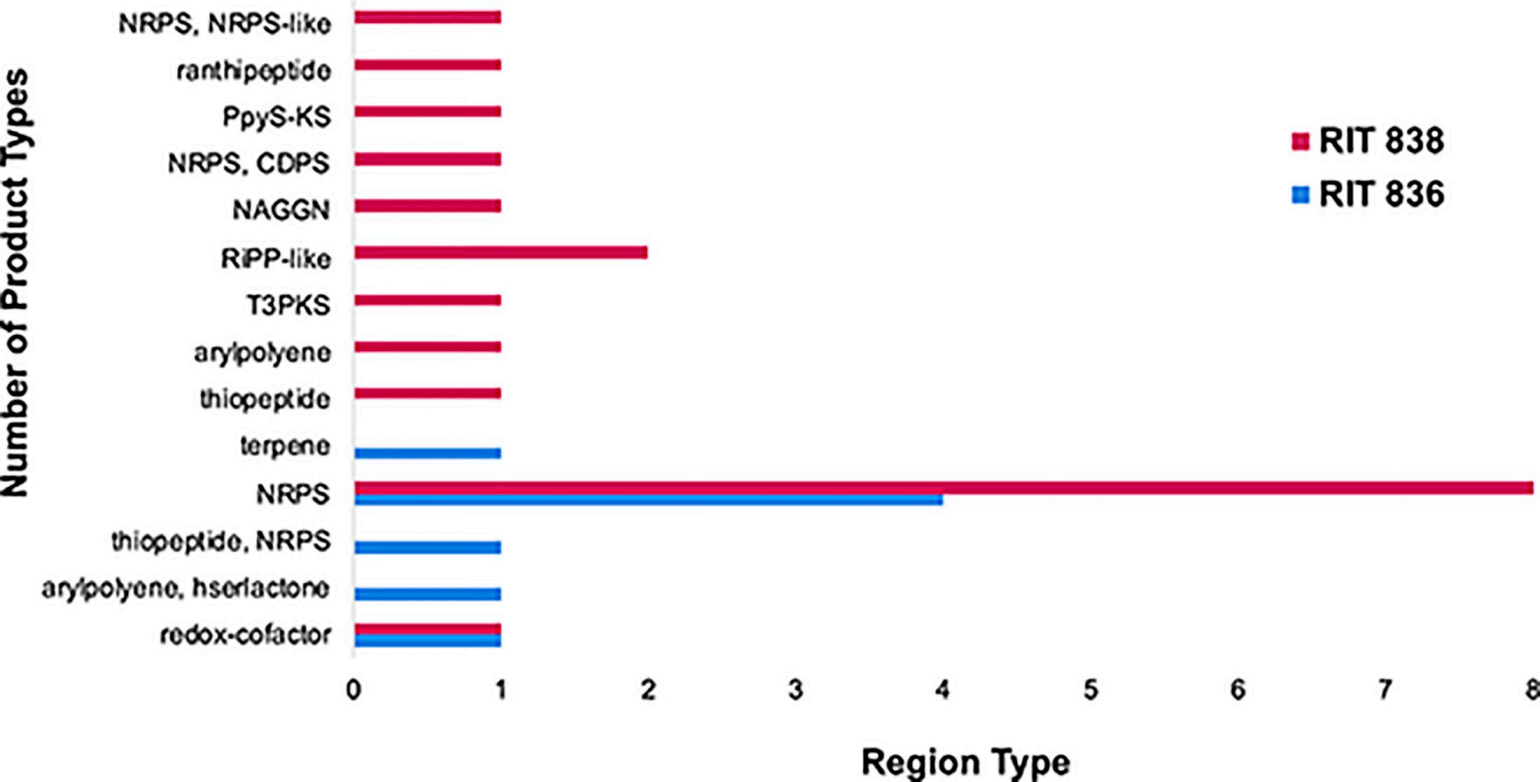
Biosynthetic gene clusters (BGC) product types detected by antiSMASH 6.1.1 analysis. Five different product types were identified in *P*. *rodasii* RIT 836 and eleven in *P*. *endophytica* RIT 838. NRPS: nonribosomal peptide-synthetase; T3PKS: type III PKS; RiPP-like: ribosomally synthesized and post-translationally modified peptide product; NAGGN: *N*-acetylglutaminylglutamine amid; CDPS: tRNA-dependent cyclodipeptide synthases; PpyS-KS: PPY-like pyrone.

antiSMASH identified five different product types of BGCs in the RIT 836 genome and eleven in the RIT 838 genome (Fig 4, S3 Table). Five out of eight regions identified from the RIT 836 genome resulted in similarity percentages of genes within the closest known compound with a significant BLAST hit within the current region. The three other regions are not similar to any known gene clusters and will need to be further studied for identification. Sixteen out of twenty regions identified from the RIT 838 genome resulted in similarity percentages of genes within the closest known compound with a significant BLAST hit within the current region. The four other regions are not similar to any known gene clusters and will need to be further studied for identification.

**Fig 5:**
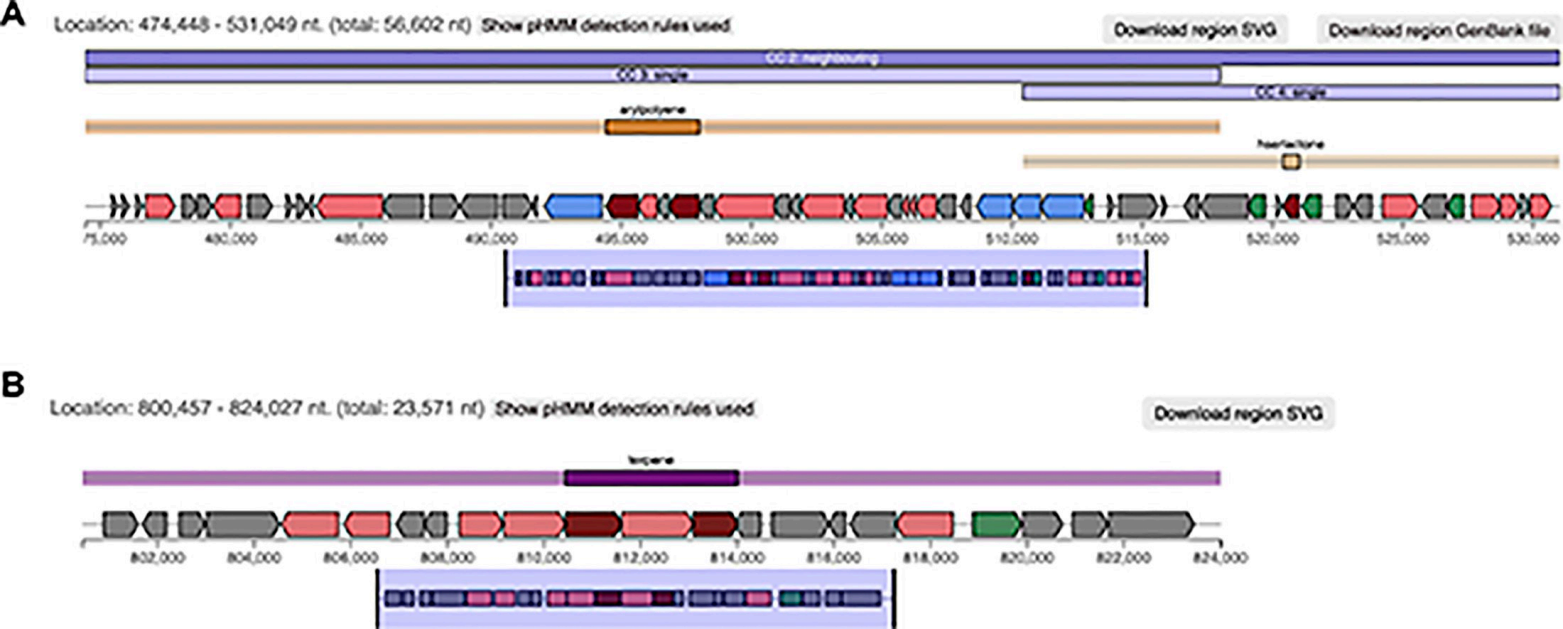
antiSMASH 6.1.1 analysis of the *P*. *rodasii* RIT 836 genome. Region 1.2 of RIT 836’s genome contains an aryl polyene-related BGC (**A**) and region 2.2 of RIT 838’s genome contains a terpene-related BGC (**B**). Cluster 2 of Region 1 (474,448–531,049 nt. within Region 1) is 94% identical to gene clusters known from *Xenorhabdus doucetiae*. Cluster 2 of Region 2 (800,457–824,027 nt. within Region 1) is 100% identical to gene clusters known from *Enterobacteriaceae bacterium* DC260. Legend: core biosynthetic genes = dark red; additional biosynthetic genes = rose-red; regulatory genes = green; transport related genes = blue; unknown function = dark grey; and resistance = light grey.

**Fig 6:**
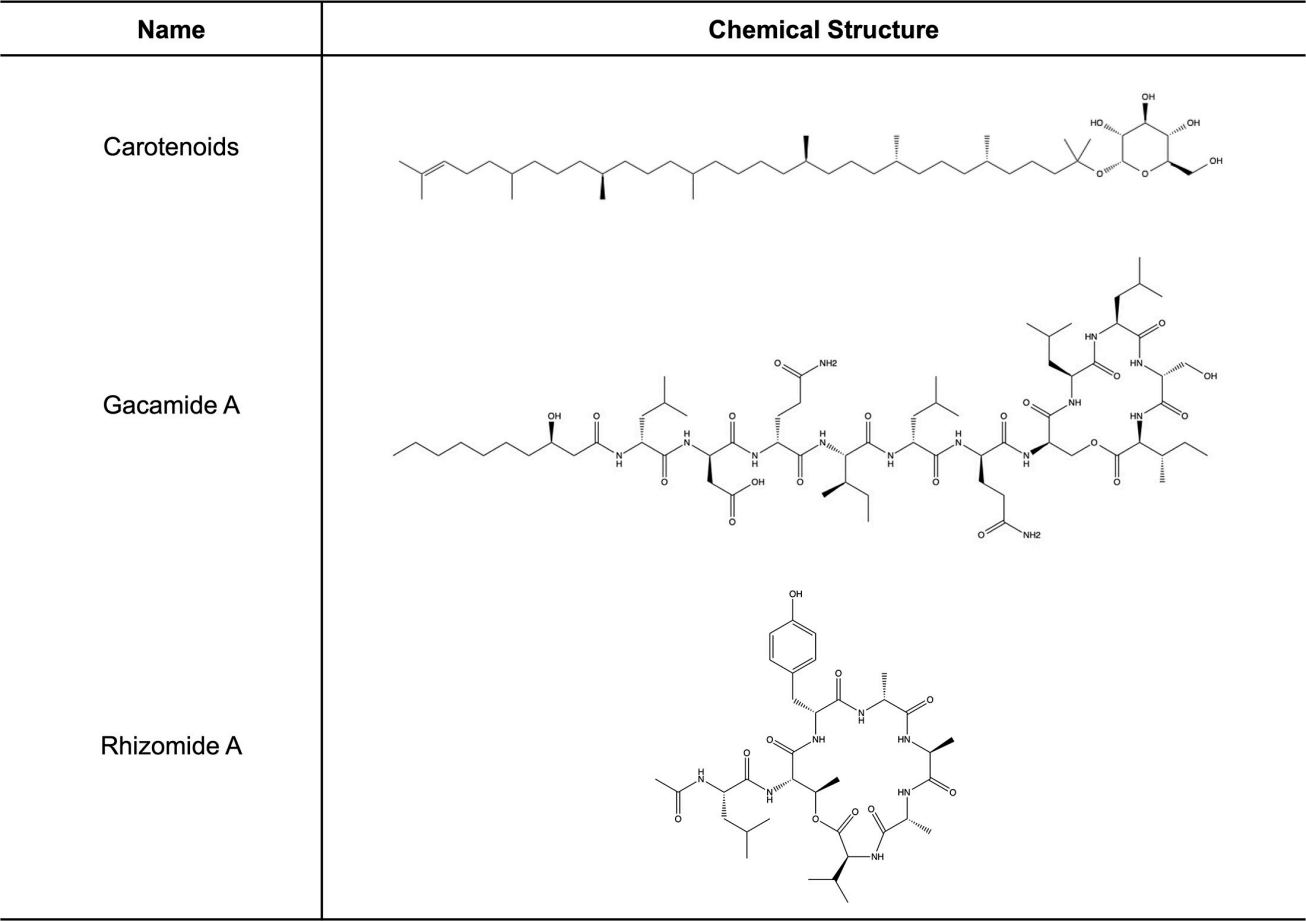
Chemical structures of compounds identified from the genome analysis of *P*. *rodasii* RIT 836 and *P*. *endophytica* RIT 838 with reported antibiotic and antibacterial activity.

Regions 2.2 from the RIT 836 genome resulted in a hit to carotenoids, which have been reported to have antibacterial activity (S4 Table, Figs 5 and 6) [51]. Carotenoids are organic, lipophilic, naturally-occurring terpenoid compounds that can range in color: yellow, orange, or red (410–510 nm) [52], which might explain the yellow-orange color of this bacterium’s colonies observed on agar plates and contribute to the antibiotic activity showed by RIT 836 in our inhibitory assays. Terpenes have the potential to inhibit microbes through molecular mechanisms involved in anti-quorum sensing, membrane disruption, and protein synthesis inhibition [53, 54]. Carotenoids extracted from strains of *Rhodotorula glutinis* have been found to have antioxidant and antibacterial effects as a natural preservative [51]. Fucoxanthin, another carotenoid, has been reported to have antibiotic activity against both aerobic and anerobic gram-positive bacteria [55].

**Fig 7:**
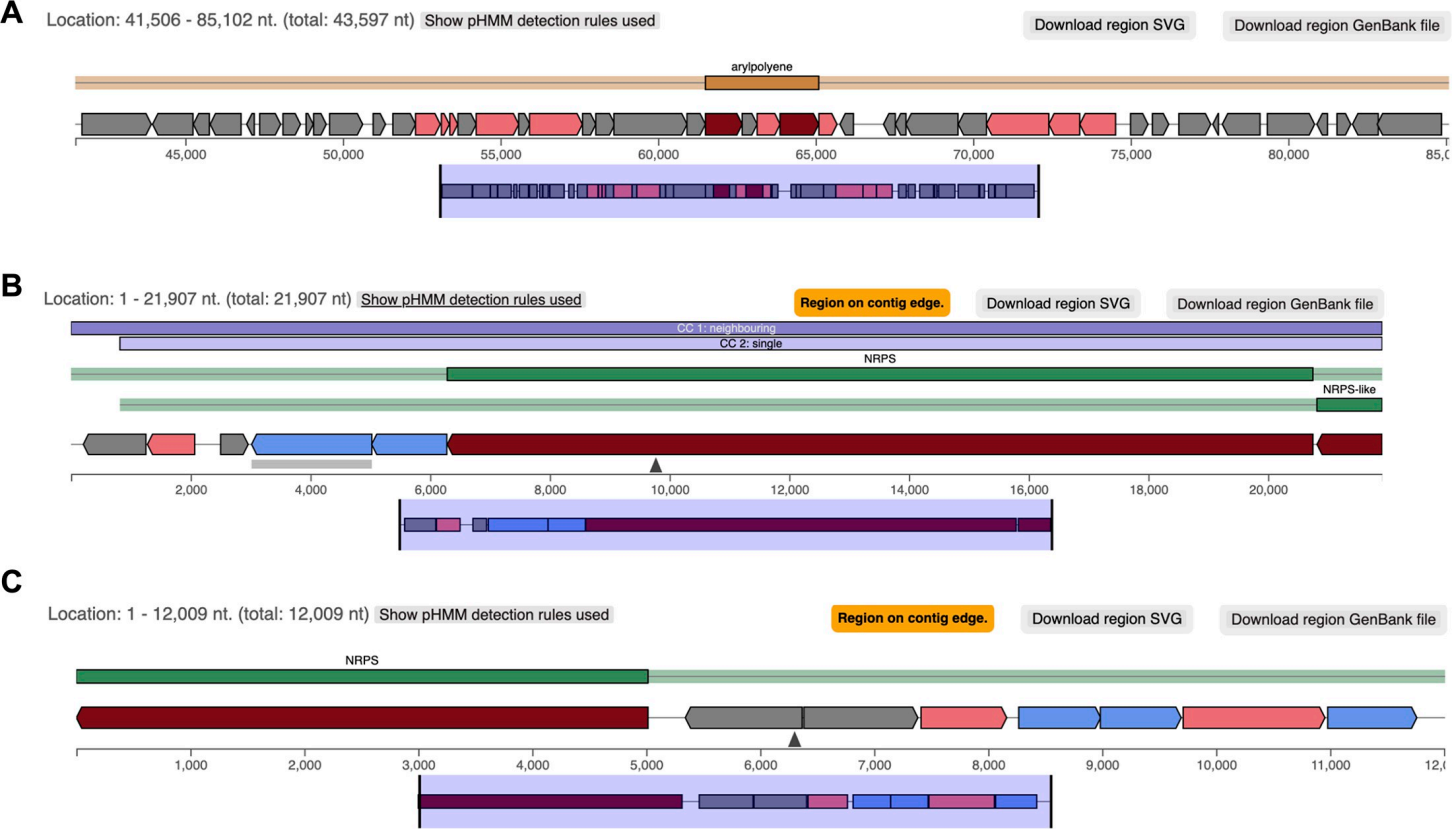
antiSMASH 6.1.1 analysis of the *P*. *endophytica* RIT 838 genome. Region 8.1 of RIT 886’s genome contains an aryl polyene-related BGC (**A**), Region 80.1 contains a NRPS, NRPS-like related BGC (**B**); and Region 92.2 contains an NRPS-related BGC (**C**). Cluster 1 of Region 8 (1,506–85,102 nt. within Region 8) is 89% identical to gene clusters known from *Escherichia coli* CFT073. Cluster 1 of Region 80 (1–21,907 nt nt. within Region 80) is 85% identical to gene clusters known from *Pseudomonas fluorescens Pf0-1*. Cluster 2 of Region 92 (1–12,009 nt. within Region 92) is 100% identical to gene clusters known from *Paraburkholderia rhizoxinica* HKI 454. Legend: core biosynthetic genes = dark red; additional biosynthetic genes = rose-red; regulatory genes = green; transport related genes = blue; unknown function = dark grey; and resistance = light grey.

Regions 1.2 from the RIT 836 genome and 8.1 from the RIT 838 genome resulted in hits to aryl polyenes (APEs), which have also been reported to increase protection from oxidative stress and contribute to biofilm formation (S4 Table, Figs 5 and 7), which might contribute to the observed antibiotic resistance shown by the two strains in our susceptibility assays [56, 57]. APEs are the product of the most extensive family of BGCs [56, 57]. Specifically, APE in *E*. *coli* (APE_EC_) is proposed to cause changes in regulatory cascades or cell envelope composition that increase biofilm formation [56]. Biofilms are formed as a part of the default defense mechanism to allow the bacteria to maintain a favorable environment, retain nutrients, and survive [58, 59]. Biofilms can tolerate antimicrobial agents but can become susceptible to antibiotic treatment when the biofilm is disrupted [59].

Regions 80.1 and 92.1 from the RIT 838 genome resulted in hits to nonribosomal peptides (NRPs) (S4 Table). NPRs are low molecular weight bioactive secondary metabolites synthesized by a ribosome-independent pathway [60]. NPRs contain non-proteinogenic amino acids, like ornithine, and can exhibit antibiotic activity [60–63], which might contribute to the antibiotic activity showed by RIT 838 in our inhibitory assays. Gacamide A, which was identified from region 80.1 from the RIT 838 genome, is a NRP:lipopeptide (Fig 6 and S4 Table) [64]. It has been shown to have moderate, narrow-spectrum antibiotic activity, and to facilitate bacterial surface motility [64]. Several lipopeptides have been reported to exhibit significant antibacterial activity [64, 65]. Rhizomide A (identified from region 92.1 from the RIT 838 genome) has reported antibacterial activity against several clinically relevant strains, including *S*. *aureus* and *B*. *subtills* (Fig 6 and S4 Table) [66–68].

We did a search on the available genomes in the Integrated Microbial Genomes and Microbiomes database and identified three *Pantoea rodasii* genomes and one *Pseudomonas endophytica* genome. We then used antiSMASH (7.0.1) to identify secondary metabolite BGCs present in these strains (S5 Table) to compare with the strains we identified in this study. All three strains of *P*. *rodasii* (DSM 26611, LMG 26273, and ND03) had a region with the most similar known cluster to APE_EC_ and another region with a most similar known cluster to carotenoid similar to the two regions found in RIT 836. All three strains of *P*. *rodasii* had 100% similarity to a carotenoid BGC from *Enterobacteriaceae bacterium* DC260 (MIBiG accession number: BGC0000639). Additionally, these three strains showed 88% similarity to an aryl polyenes BGC from *Xenorhabdus doucetiae* (MIBiG accession number: BGC0002008). In contrast, *P*. *endophytica* BSTT44 did not present any regions with similar known clusters to RIT 838.

## Conclusions

Herein, we reported the isolation and characterization of two strains of antibiotic -producing and -resistant bacteria, *P*. *rodasii* RIT 836 and *P*. *endophytica* RIT 838, that were obtained from diverse environmental samples within a university campus. These bacteria, along with the additional strains we isolated from our samples, showed resistance to multiple commonly used antibiotics. It is important to mention that our screening conditions lacked anaerobic culture, which prevented the identification of additional bacteria in our study, particularly from soil. These findings, nonetheless, are still alarming since they confirm the presence of highly resistant bacteria that share the same environment with humans and serve as another warning for the importance of fighting the spread of AMR. The two strains highlighted in this study also demonstrated the ability to produce bactericidal activity against both Gram-positive and Gram-negative species. RIT 838, in particular, showed the most significant broad-spectrum antibiotic activity, making this bacterium of great interest for further studies.

Antibiotics fall under the category of a secondary metabolite and can be used as a defensive mechanism against other microorganisms [69]. Secondary metabolites are not directly related to the growth of a microorganism [49]. About 70% of anti-infective drugs that are used in medicine have been derived from natural products [49]. It is essential to continue to screen microorganisms for molecules that can be used as antimicrobial agents to help aid in the antibiotic resistant crisis [52]. According to our genome annotation and analysis of both RIT 836 and RIT 838 bacteria, we predicted various regions of secondary metabolite BGCs that are potentially responsible for antibiotic production in these bacteria. In future work, we plan to isolate, identify, and fully characterize the chemical compounds produced by both species (especially RIT 838) that are endowed with antimicrobial activity, as well as test these compounds against clinical multidrug resistant isolates, and investigate their potential targets and mechanisms of action.

## Supporting information

S1 File (DOCX)

## References

[1] About Antimicrobial Resistance. [cited 13 Feb 2023]. https://www.cdc.gov/drugresistance/about.html.

[2] Matlock A, Garcia JA, Moussavi K, Long B, Liang SY-T. Advances in novel antibiotics to treat multidrug-resistant gram-negative bacterial infections. Internal and Emergency Medicine. 2021;16: 2231–2241. doi: 10.1007/s11739-021-02749-1 33956311 PMC8100742

[3] de Kraker MEA, Stewardson AJ, Harbarth S. Will 10 Million People Die a Year due to Antimicrobial Resistance by 2050? PLOS Medicine. 2016;13: e1002184. doi: 10.1371/journal.pmed.1002184 27898664 PMC5127510

[4] Murray CJ, Ikuta KS, Sharara F, Swetschinski L, Robles Aguilar G, Gray A, et al. Global burden of bacterial antimicrobial resistance in 2019: a systematic analysis. The Lancet. 2022;399: 629–655. doi: 10.1016/S0140-6736(21)02724-0 35065702 PMC8841637

[5] Antimicrobial Resistance. [cited 13 Feb 2023].https://www.who.int/news-room/fact-sheets/detail/antimicrobial-resistance.

[6] Antibiotics. [cited 13 Feb 2023]. https://medlineplus.gov/antibiotics.html.

[7] Lambert P. Bacterial resistance to antibiotics: Modified target sites. Advanced Drug Delivery Reviews. 2005;57: 1471–1485. doi: 10.1016/j.addr.2005.04.003 15964098

[8] Tenover FC. Mechanisms of antimicrobial resistance in bacteria. American Journal of Infection Control. 2006;34: S3–S10. doi: 10.1016/j.ajic.2006.05.219 16813980

[9] Coates A, Hu Y, Bax R, Page C. The future challenges facing the development of new antimicrobial drugs. Nature Reviews Drug Discovery. 2002;1: 895–910. doi: 10.1038/nrd940 12415249

[10] Park JT, Strominger JL. Mode of Action of Penicillin. Science. 1957;125: 99–101. doi: 10.1126/science.125.3238.99 13390969

[11] Shockman GD, Daneo-Moore L, Cornett JB, Mychajlonkat M. Does Penicillin Kill Bacteria? Clinical Infectious Diseases. 1979;1: 787–796. doi: 10.1093/clinids/1.5.787 44383

[12] Kapoor G, Saigal S, Elongavan A. Action and resistance mechanisms of antibiotics: A guide for clinicians. Journal of Anaesthesiology Clinical Pharmacology. 2017;33: 300. doi: 10.4103/joacp.JOACP_349_15 29109626 PMC5672523

[13] Bhattacharjee MK. Antibiotics That Inhibit Nucleic Acid Synthesis. Chemistry of Antibiotics and Related Drugs. 2016; 109–128. doi: 10.1007/978-3-319-40746-3_5

[14] Antibiotic Resistance. [cited 13 Feb 2023]. https://www.cdc.gov/ncezid/what-we-do/2021-highlights/antibiotic-resistance.html.

[15] Larsson DGJ, Flach C-F. Antibiotic resistance in the environment. Nature Reviews Microbiology. 2022;20: 257–269. doi: 10.1038/s41579-021-00649-x 34737424 PMC8567979

[16] Golkar Z, Bagasra O, Pace DG. Bacteriophage therapy: a potential solution for the antibiotic resistance crisis. The Journal of Infection in Developing Countries. 2014;8: 129–136. doi: 10.3855/jidc.3573 24518621

[17] Singh SB, Barrett JF. Empirical antibacterial drug discovery—Foundation in natural products. Biochemical Pharmacology. 2006;71: 1006–1015. doi: 10.1016/j.bcp.2005.12.016 16412984

[18] C Reygaert W. An overview of the antimicrobial resistance mechanisms of bacteria. AIMS Microbiology. 2018;4: 482–501. doi: 10.3934/microbiol.2018.3.482 31294229 PMC6604941

[19] Baker S, Thomson N, Weill F-X, Holt KE. Genomic insights into the emergence and spread of antimicrobial-resistant bacterial pathogens. Science. 2018;360: 733–738. doi: 10.1126/science.aar3777 29773743 PMC6510332

[20] How Antimicrobial Resistance Happens. [cited 13 Feb 2023]. https://www.cdc.gov/drugresistance/about/how-resistance-happens.html.

[21] Levy SB, Marshall B. Antibacterial resistance worldwide: causes, challenges and responses. Nature Medicine. 2004;10: S122–S129. doi: 10.1038/nm1145 15577930

[22] Davies J, Davies D. Origins and Evolution of Antibiotic Resistance. Microbiology and Molecular Biology Reviews. 2010;74: 417–433. doi: 10.1128/MMBR.00016-10 20805405 PMC2937522

[23] Conly JM, Johnston BL. Where are all the new antibiotics? The new antibiotic paradox. Canadian Journal of Infectious Diseases and Medical Microbiology. 2005;16: 159–160. doi: 10.1155/2005/892058 18159536 PMC2095020

[24] Powers JH. Antimicrobial drug development—the past, the present, and the future. Clinical Microbiology and Infection. 2004;10: 23–31. doi: 10.1111/j.1465-0691.2004.1007.x 15522037

[25] Miranda RR, Parthasarathy A, Hudson AO. Exploration of Chemical Biology Approaches to Facilitate the Discovery and Development of Novel Antibiotics. Frontiers in Tropical Diseases. 2022;3. doi: 10.3389/fitd.2022.845469

[26] Lewis K. The Science of Antibiotic Discovery. Cell. 2020;181: 29–45. doi: 10.1016/j.cell.2020.02.056 32197064

[27] Lewis K. Platforms for antibiotic discovery. Nature Reviews Drug Discovery. 2013;12: 371–387. doi: 10.1038/nrd3975 23629505

[28] Fernandes P. Antibacterial discovery and development—the failure of success? Nature Biotechnology. 2006;24: 1497–1503. doi: 10.1038/nbt1206-1497 17160049

[29] Mantravadi P, Kalesh K, Dobson R, Hudson A, Parthasarathy A. The Quest for Novel Antimicrobial Compounds: Emerging Trends in Research, Development, and Technologies. Antibiotics. 2019;8: 8. doi: 10.3390/antibiotics8010008 30682820 PMC6466574

[30] Coates AR, Halls G, Hu Y. Novel classes of antibiotics or more of the same? British Journal of Pharmacology. 2011;163: 184–194. doi: 10.1111/j.1476-5381.2011.01250.x 21323894 PMC3085877

[31] Miethke M, Pieroni M, Weber T, Brönstrup M, Hammann P, Halby L, et al. Towards the sustainable discovery and development of new antibiotics. Nature Reviews Chemistry. 2021;5: 726–749. doi: 10.1038/s41570-021-00313-1 34426795 PMC8374425

[32] Antibiotic resistance threats in the United States, 2019. 10.15620/cdc:82532.

[33] National Infection & Death Estimates. [cited 13 Feb 2023]. https://www.cdc.gov/drugresistance/national-estimates.html.

[34] Michael CA, Dominey-Howes D, Labbate M. The Antimicrobial Resistance Crisis: Causes, Consequences, and Management. Frontiers in Public Health. 2014;2. doi: 10.3389/fpubh.2014.00145 25279369 PMC4165128

[35] Cycoń M, Mrozik A, Piotrowska-Seget Z. Antibiotics in the Soil Environment—Degradation and Their Impact on Microbial Activity and Diversity. Front Microbiol. 2019;10: 338. doi: 10.3389/fmicb.2019.00338 30906284 PMC6418018

[36] Daghrir R, Drogui P. Tetracycline antibiotics in the environment: a review. Environ Chem Lett. 2013;11: 209–227. doi: 10.1007/s10311-013-0404-8

[37] The “Three Cs” of Novel Antibiotic Discovery and Production through Synthetic Biology: Biosynthetic Gene Clusters, Heterologous Chassis, and Synthetic Microbial Consortia—Baker—2018—Advanced Biosystems—Wiley Online Library. [cited 10 Apr 2023]. https://onlinelibrary.wiley.com/10.1002/adbi.201800064.

[38] Blin K, Shaw S, Kloosterman AM, Charlop-Powers Z, van Wezel GP, Medema MH, et al. antiSMASH 6.0: improving cluster detection and comparison capabilities. Nucleic Acids Res. 2021;49: W29–W35. doi: 10.1093/nar/gkab335 33978755 PMC8262755

[39] Wick RR, Judd LM, Gorrie CL, Holt KE. Unicycler: Resolving bacterial genome assemblies from short and long sequencing reads. PLOS Computational Biology. 2017;13: e1005595. doi: 10.1371/journal.pcbi.1005595 28594827 PMC5481147

[40] Bankevich A, Nurk S, Antipov D, Gurevich AA, Dvorkin M, Kulikov AS, et al. SPAdes: A New Genome Assembly Algorithm and Its Applications to Single-Cell Sequencing. Journal of Computational Biology. 2012;19: 455–477. doi: 10.1089/cmb.2012.0021 22506599 PMC3342519

[41] Gurevich A, Saveliev V, Vyahhi N, Tesler G. QUAST: quality assessment tool for genome assemblies. Bioinformatics. 2013;29: 1072–1075. doi: 10.1093/bioinformatics/btt086 23422339 PMC3624806

[42] Parthasarathy A. Scanning electron microscopy (SEM) for microbes -a simple and inexpensive method for sample preparation Identification of Bacteria on Smartphone screens View project. 2019. doi: 10.13140/RG.2.2.26222.05449

[43] Hentges David J. Anaerobes: General Characteristics. 4th ed. In: Baron Samuel, editor. Medical microbiology. 4th ed. Galveston, Tex: University of Texas Medical Branch at Galveston; 1996. https://www.ncbi.nlm.nih.gov/books/NBK7638/. 21413255

[44] Walterson AM, Stavrinides J. Pantoea: insights into a highly versatile and diverse genus within the Enterobacteriaceae. FEMS Microbiology Reviews. 2015;39: 968–984. doi: 10.1093/femsre/fuv027 26109597

[45] Moradali MF, Ghods S, Rehm BHA. Pseudomonas aeruginosa Lifestyle: A Paradigm for Adaptation, Survival, and Persistence. Frontiers in Cellular and Infection Microbiology. 2017;7. https://www.frontiersin.org/articles/10.3389/fcimb.2017.00039. 28261568 10.3389/fcimb.2017.00039 PMC5310132

[46] Brodey CL. Bacterial Blotch Disease of the Cultivated Mushroom Is Caused by an Ion Channel Forming Lipodepsipeptide Toxin. MPMI. 1991;4: 407. doi: 10.1094/MPMI-4-407

[47] Mulani MS, Kamble EE, Kumkar SN, Tawre MS, Pardesi KR. Emerging Strategies to Combat ESKAPE Pathogens in the Era of Antimicrobial Resistance: A Review. Frontiers in Microbiology. 2019;10. Available: https://www.frontiersin.org/articles/10.3389/fmicb.2019.00539. 30988669 10.3389/fmicb.2019.00539 PMC6452778

[48] Medema MH, Blin K, Cimermancic P, de Jager V, Zakrzewski P, Fischbach MA, et al. antiSMASH: rapid identification, annotation and analysis of secondary metabolite biosynthesis gene clusters in bacterial and fungal genome sequences. Nucleic Acids Res. 2011;39: W339–W346. doi: 10.1093/nar/gkr466 21672958 PMC3125804

[49] Chen R, Wong HL, Kindler GS, MacLeod FI, Benaud N, Ferrari BC, et al. Discovery of an Abundance of Biosynthetic Gene Clusters in Shark Bay Microbial Mats. Frontiers in Microbiology. 2020;11. Available: https://www.frontiersin.org/articles/10.3389/fmicb.2020.01950 32973707 10.3389/fmicb.2020.01950 PMC7472256

[50] Medema MH, Kottmann R, Yilmaz P, Cummings M, Biggins JB, Blin K, et al. Minimum Information about a Biosynthetic Gene cluster. Nat Chem Biol. 2015;11: 625–631. doi: 10.1038/nchembio.1890 26284661 PMC5714517

[51] Keceli TM, Erginkaya Z, Turkkan E, Kaya U. Antioxidant and Antibacterial Effects of Carotenoids Extracted from Rhodotorula glutinis Strains. Asian J Chem. 2013;25: 42–46. doi: 10.14233/ajchem.2013.12377

[52] Vargas-Sinisterra AF, Ramírez-Castrillón M. Yeast carotenoids: production and activity as antimicrobial biomolecule. Arch Microbiol. 2021;203: 873–888. doi: 10.1007/s00203-020-02111-7 33151382

[53] Li H, Maimaitiming M, Zhou Y, Li H, Wang P, Liu Y, et al. Discovery of Marine Natural Products as Promising Antibiotics against Pseudomonas aeruginosa. Mar Drugs. 2022;20: 192. doi: 10.3390/md20030192 35323491 PMC8954164

[54] Sharma A, Biharee A, Kumar A, Jaitak V. Antimicrobial Terpenoids as a Potential Substitute in Overcoming Antimicrobial Resistance. Curr Drug Targets. 2020;21: 1476–1494. doi: 10.2174/1389450121666200520103427 32433003

[55] Karpiński TM, Adamczak A. Fucoxanthin—An Antibacterial Carotenoid. Antioxidants (Basel). 2019;8: 239. doi: 10.3390/antiox8080239 31344844 PMC6720875

[56] Johnston I, Osborn LJ, Markley RL, McManus EA, Kadam A, Schultz KB, et al. Identification of essential genes for Escherichia coli aryl polyene biosynthesis and function in biofilm formation. npj Biofilms Microbiomes. 2021;7: 1–10. doi: 10.1038/s41522-021-00226-3 34215744 PMC8253772

[57] Cimermancic P, Medema MH, Claesen J, Kurita K, Wieland Brown LC, Mavrommatis K, et al. Insights into Secondary Metabolism from a Global Analysis of Prokaryotic Biosynthetic Gene Clusters. Cell. 2014;158: 412–421. doi: 10.1016/j.cell.2014.06.034 25036635 PMC4123684

[58] Khatoon Z, McTiernan CD, Suuronen EJ, Mah T-F, Alarcon EI. Bacterial biofilm formation on implantable devices and approaches to its treatment and prevention. Heliyon. 2018;4: e01067. doi: 10.1016/j.heliyon.2018.e01067 30619958 PMC6312881

[59] Bjarnsholt T. The role of bacterial biofilms in chronic infections. APMIS. 2013;121: 1–58. doi: 10.1111/apm.12099 23635385

[60] Yadav I, Devi N, Singh S. Nonribosomal Peptide Synthesis in Microbes. Recent Advances in Microbiology. 2012. p. 183. https://www.researchgate.net/profile/Ningombam-Devi-2/publication/261570326_Microbial_degradation_of_chlorinated_aromatic_hydrocarbons/links/5554c0be08ae980ca60ad0f5/Microbial-degradation-of-chlorinated-aromatic-hydrocarbons.pdf.

[61] Srivastava AK, Srivastava R, Bharati AP, Singh AK, Sharma A, Das S, et al. Analysis of Biosynthetic Gene Clusters, Secretory, and Antimicrobial Peptides Reveals Environmental Suitability of Exiguobacterium profundum PHM11. Frontiers in Microbiology. 2022;12. https://www.frontiersin.org/articles/10.3389/fmicb.2021.785458 35185816 10.3389/fmicb.2021.785458 PMC8851196

[62] Marahiel MA. Working outside the protein-synthesis rules: insights into non-ribosomal peptide synthesis. J Pept Sci. 2009;15: 799–807. doi: 10.1002/psc.1183 19827002

[63] Steiner KK, Parthasarathy A, Wong NH, Cavanaugh NT, Chu J, Hudson AO. Isolation and whole-genome sequencing of Pseudomonas sp. RIT 623, a slow-growing bacterium endowed with antibiotic properties. BMC Research Notes. 2020;13: 370. doi: 10.1186/s13104-020-05216-w 32746897 PMC7398229

[64] Jahanshah G, Yan Q, Gerhardt H, Pataj Z, Lämmerhofer M, Pianet I, et al. Discovery of the Cyclic Lipopeptide Gacamide A by Genome Mining and Repair of the Defective GacA Regulator in Pseudomonas fluorescens Pf0-1. J Nat Prod. 2019;82: 301–308. doi: 10.1021/acs.jnatprod.8b00747 30666877

[65] Development of novel broad-spectrum antimicrobial lipopeptides derived from plantaricin NC8 β | Scientific Reports. [cited 17 Apr 2023]. https://www.nature.com/articles/s41598-023-31185-8. 10.1038/s41598-023-31185-8 PMC10011573 36914718

[66] Abdel-Mageed WM, Al-Wahaibi LH, Lehri B, Al-Saleem MSM, Goodfellow M, Kusuma AB, et al. Biotechnological and Ecological Potential of Micromonospora provocatoris sp. nov., a Gifted Strain Isolated from the Challenger Deep of the Mariana Trench. Marine Drugs. 2021;19: 243. doi: 10.3390/md19050243 33923039 PMC8146288

[67] Gavriilidou A, Mackenzie TA, Sánchez P, Tormo JR, Ingham C, Smidt H, et al. Bioactivity Screening and Gene-Trait Matching across Marine Sponge-Associated Bacteria. Marine Drugs. 2021;19: 75. doi: 10.3390/md19020075 33573261 PMC7912018

[68] Wang X, Zhou H, Chen H, Jing X, Zheng W, Li R, et al. Discovery of recombinases enables genome mining of cryptic biosynthetic gene clusters in Burkholderiales species. Proceedings of the National Academy of Sciences. 2018;115: E4255–E4263. doi: 10.1073/pnas.1720941115 29666226 PMC5939090

[69] Demain AL, Fang A. The Natural Functions of Secondary Metabolites. In: Fiechter A, editor. History of Modern Biotechnology I. Berlin, Heidelberg: Springer; 2000. pp. 1–39. doi: 10.1007/3-540-44964-7_1 11036689

