# Supplementary material for: Isolation, whole-genome sequencing, and annotation of two antibiotic-producing and antibiotic-resistant bacteria, Pantoea rodasii RIT 836 and Pseudomonas endophytica RIT 838, collected from the environment
Source: PLoS One. 2024 Feb 27;19(2):e0293943. doi: 10.1371/journal.pone.0293943 (PMC10898753; doi:10.1371/journal.pone.0293943)
Supplement: S1 File — (DOCX) [file pone.0293943.s001.docx]

**Supporting Information**

**Isolation, whole-genome sequencing, and annotation of two antibiotic-producing and antibiotic-resistant bacteria, *Pantoea rodasii* RIT 836 and *Pseudomonas endophytica* RIT 838, collected from the environment**

**Serena Tuytschaevers^1^, Leila Aden^2^, Zacchaeus Greene^2^, Chanei Nixon^2^, Wade Shaw^2^, Dillan Hatch^1^, Girish Kumar^1^, Renata Rezende Miranda^3,^*, and André O. Hudson^1,^***

^1^ Thomas H. Gosnell School of Life Sciences, Rochester Institute of Technology, Rochester, New York, USA

^2^ Rochester Prep High School, Rochester, New York, USA

^3^ School of Chemistry and Materials Science, Rochester Institute of Technology, Rochester, New York, USA

***** Correspondence: rrmsbi@rit.edu; aohsbi@rit.edu

**Table of Contents**

**Fig S1.** Disc-diffusion susceptibility assays of eleven bacterial strains isolated from the environment around the RIT campus…...............................................................................................................…………………………..S3

**Fig S2.** Disc-diffusion inhibitory assays of *P. rodasii* RIT 836 spent LB medium extracts tested against various bacteria…...............................................................................................................……………………………….S4

**Fig S3.** Disc-diffusion inhibitory assays of *P. endophytica* RIT 838 spent LB medium extracts tested against various bacteria.................................................................................................................……………………….S5

**Table S1.** Zone of inhibition (ZOI) values measured from disc-diffusion susceptibility assays of eleven bacterial strains isolated from the environment around the RIT campus when treated with several antibiotics..….........................................................................................................................…………………....S6

**Table S2.** Zone of inhibition (ZOI) values measured from disc-diffusion inhibitory assays of *P. rodasii* RIT 836 and *P. endophytica* RIT 838 spent LB medium extracts tested against various bacteria.………………………....S6

**Table S3.** Summary of all biosynthetic gene cluster regions found in *P. rodasii* RIT 836 and *P. endophytica* RIT 838 antiSMASH analysis………………...............................................................................................................S7

**Table S4.** MIBiG Comparison, ClusterBlast analysis, and KnownClusterBlast analysis of the genomes of *P. rodasii* RIT 836 and *P. endophytica* RIT 838…………………………………………………..……..…………….….S9

**Table S5.** Summary of all biosynthetic gene cluster regions found in *P. rodasii* ND03, *P. rodasii* LMG 26273, *P. rodasii* DSM 26611, and *P. endophytica* BSTT44 antiSMASH analysis………………………………….…….….S15

**
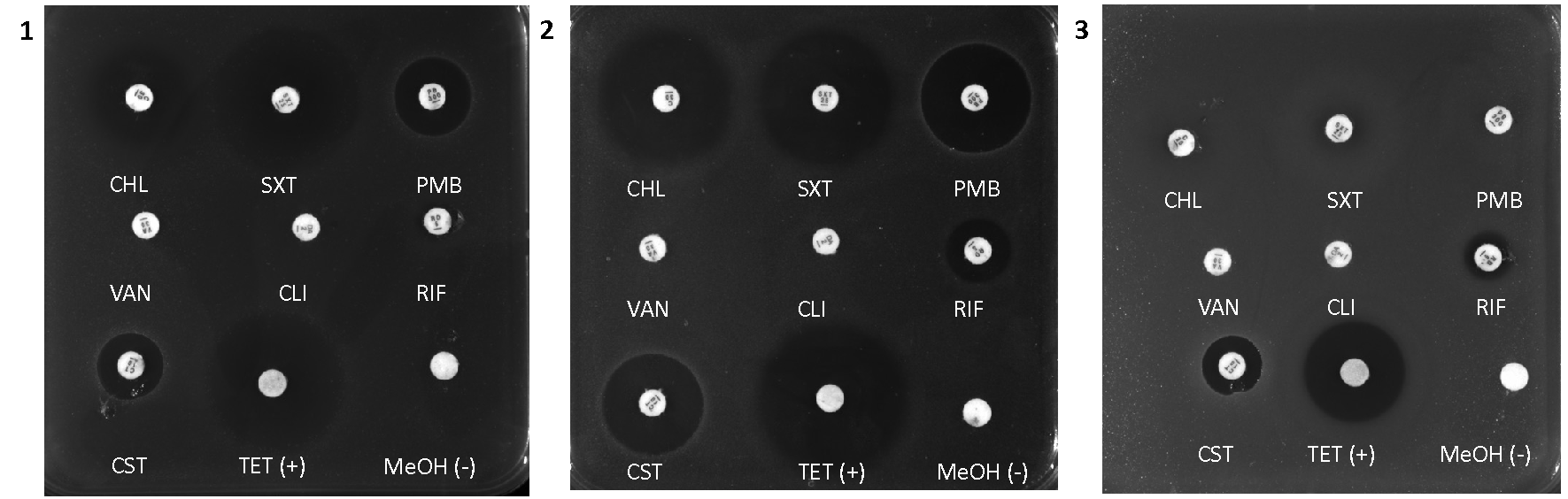
**

**
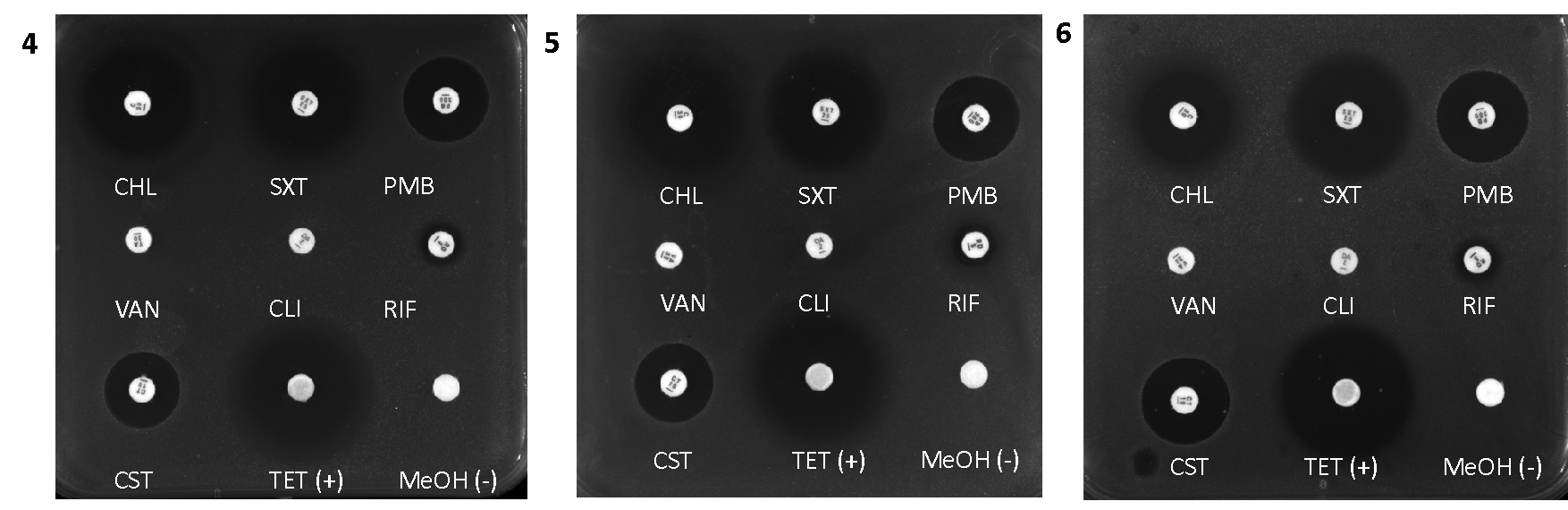
**

**
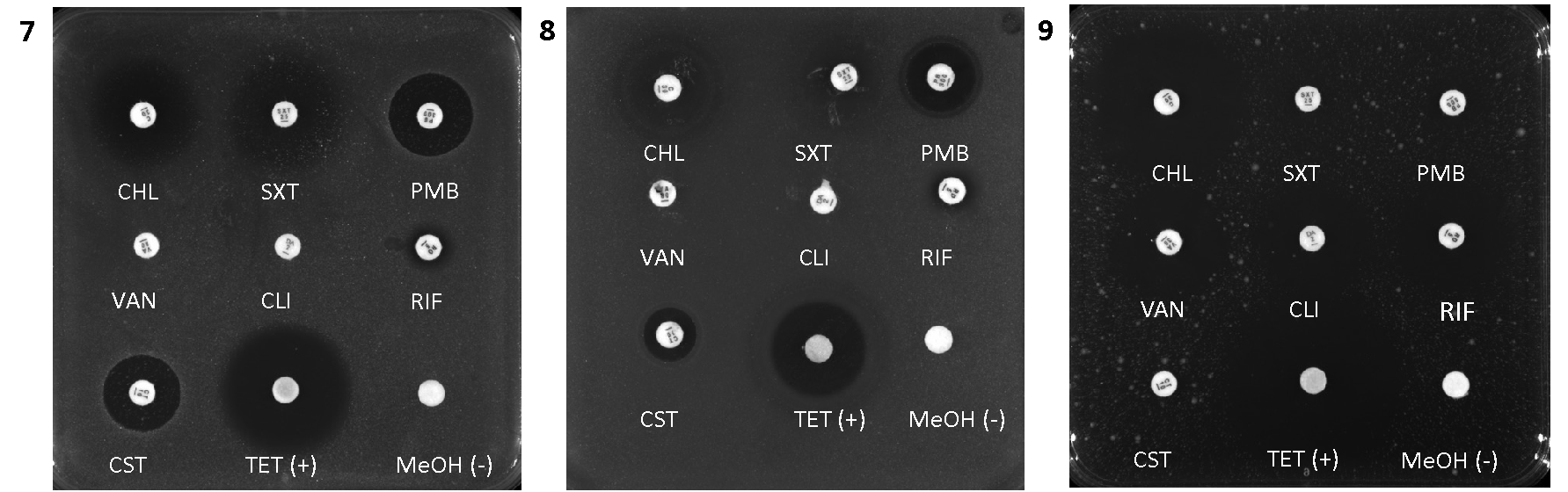
**

**
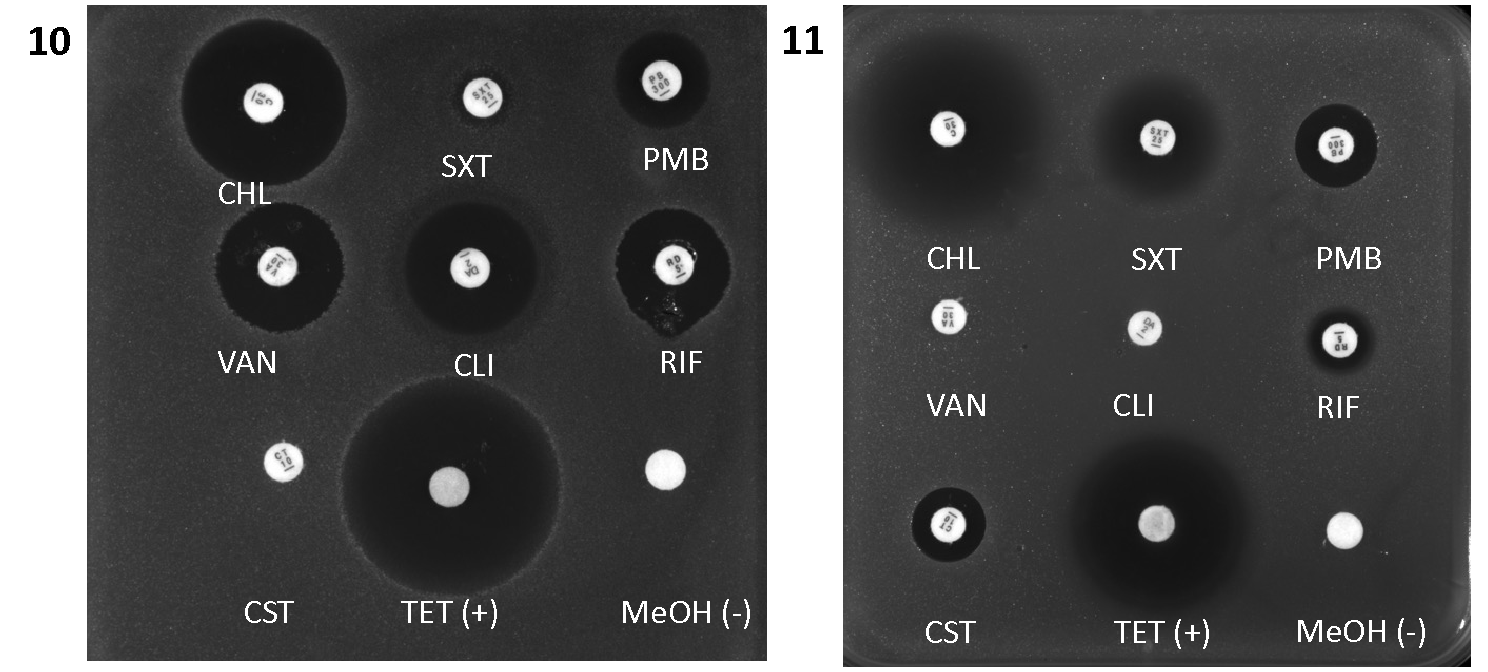
**

**Fig S1.** Disc-diffusion susceptibility assays of eleven bacterial strains isolated from the environment around the RIT campus, each treated with chloramphenicol, 30 µg (CHL); sulfamethoxazole/trimethoprim, 25 µg (SXT); polymyxin B, 300 IU (PMB); vancomycin, 30 µg (VAN); clindamycin, 2 µg (CLI); rifampicin, 5 µg (RIF); colistin sulfate, 10 µg (CST); tetracycline, 200 µg (TET, +); and methanol, 10 µL (MeOH, -). These experiments were performed in duplicates and the average ZOI values are shown in Table S1.


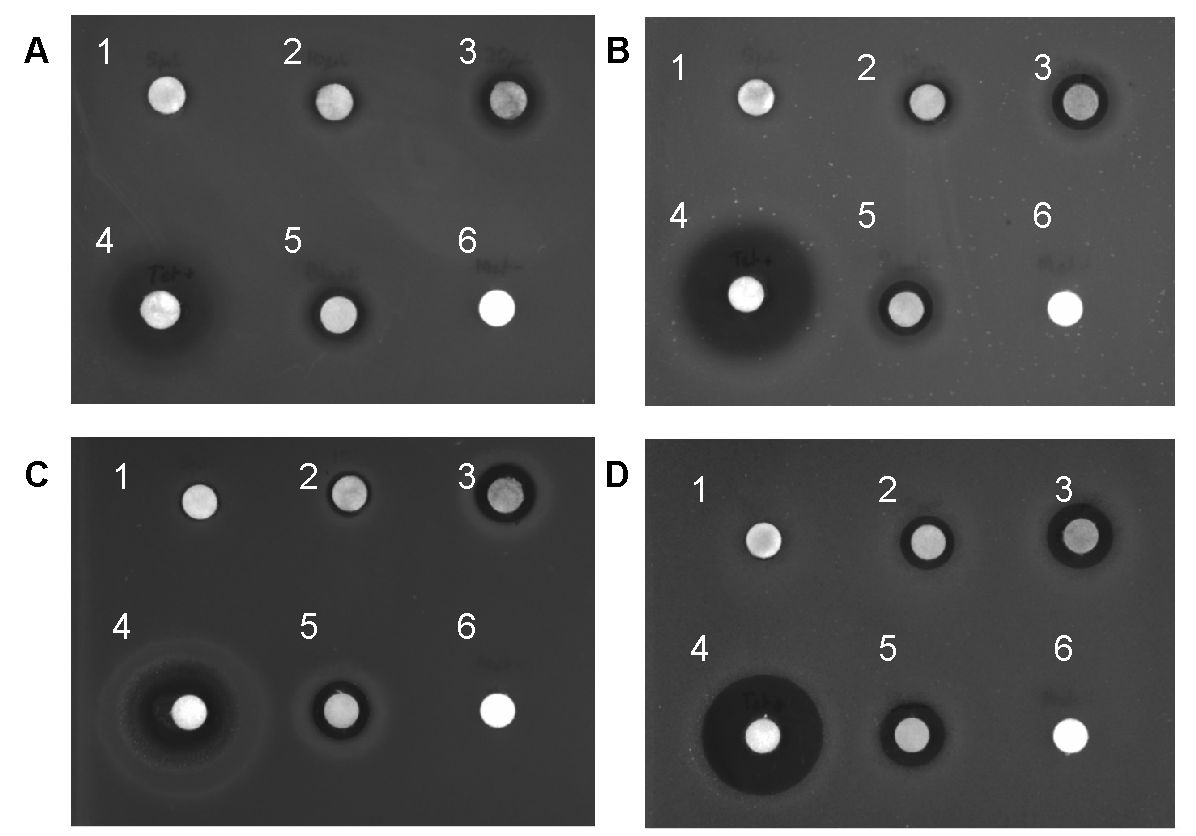


**Fig S2.** Disc-diffusion inhibitory assays of *P. rodasii* RIT 836 spent LB medium extracts tested against various bacteria: *Escherichia coli* (**a**), *Staphylococcus aureus* (**b**), *Pseudomonas aeruginosa* (**c**), and *Bacillus subtilis*(**d**). Disc contents: 1,250 µg (1), 2,500 µg (2), and 5,000 µg (3) of bacterial spent LB medium extract crude; tetracycline 200 µg (4); 5,000 µg (5) of blank LB medium (no bacteria) extract crude; and methanol, 20 µL (6).


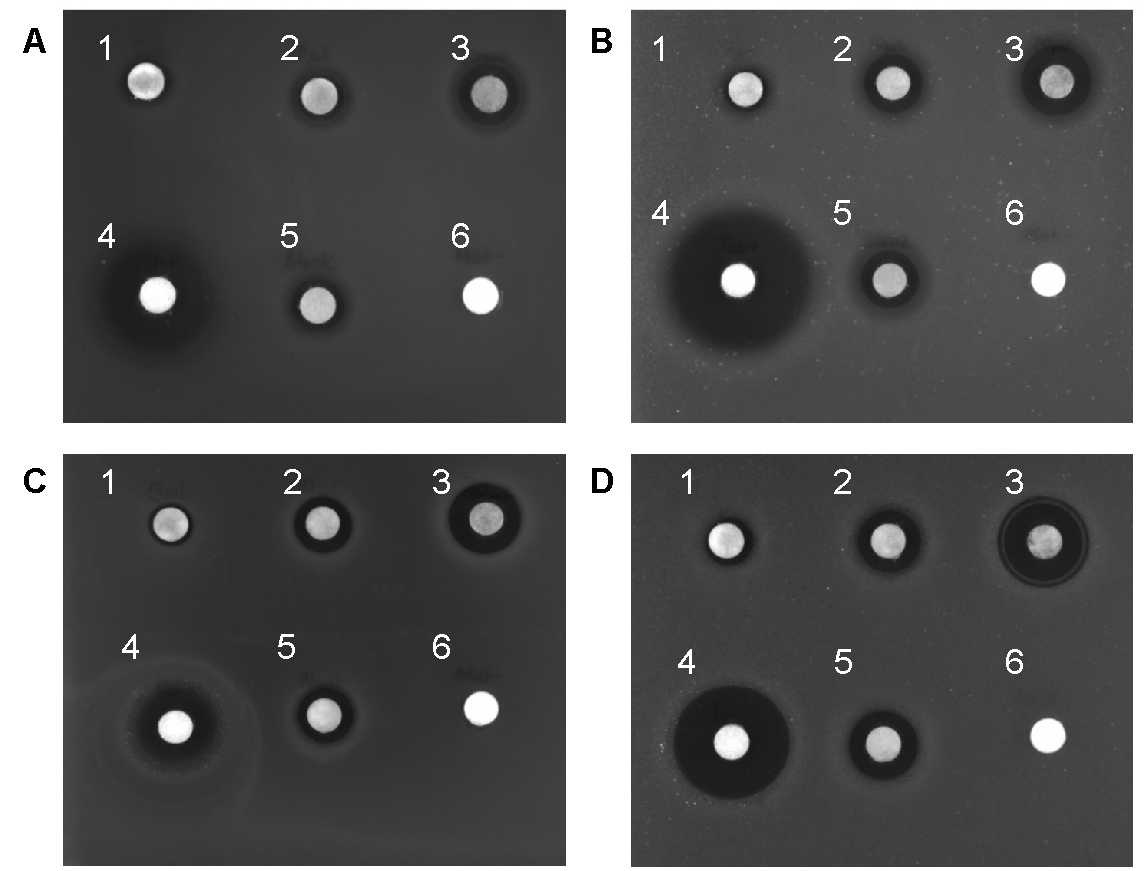


**Fig S3.** Disc-diffusion inhibitory assays of *P. endophytica* RIT 838 spent LB medium extracts tested against various bacteria: *Escherichia coli* (**a**), *Staphylococcus aureus* (**b**), *Pseudomonas aeruginosa* (**c**), and *Bacillus subtilis*(**d**). Disc contents: 1,250 µg (1), 2,500 µg (2), and 5,000 µg (3) of bacterial spent LB medium extract crude; tetracycline 200 µg (4); 5,000 µg (5) of blank LB medium (no bacteria) extract crude; and methanol, 20 µL (6).

**Table S1.** Zone of inhibition (ZOI) values measured from disc-diffusion susceptibility assays of eleven bacterial strains isolated from the environment around the RIT campus when treated with several antibiotics.

| **Conditions/Strain** | | **1** | **2** | **3*** | **4** | **5** | **6** | **7** | **8*** | **9** | **10** | **11** |
| --- | --- | --- | --- | --- | --- | --- | --- | --- | --- | --- | --- | --- |
| **Antibiotic** | **Amount** | **ZOI (mm)** | | | | | | | | | | |
| **CHL** | **30 µg** | 27.2 ± 1.09 | 28.3 ± 0.584 | 0.000 ± 0.000 | 26.3 ± 1.37 | 28.5 ± 0.780 | 20.3 ± 0.285 | 14.0 ± 0.861 | 0.000 ± 0.000 | 33.0 ± 1.66 | 25.2 ± 0.262 | 30.0 ± 0.848 |
| **SXT** | **25 µg** | 23.2 ± 0.521 | 27.4 ± 0.558 | 0.000 ± 0.000 | 25.3 ± 0.920 | 28.2 ± 0.666 | 25.7 ± 0.463 | 11.2 ± 0.517 | 0.000 ± 0.000 | 0.000 ± 0.000 | 7.31 ± 0.226 | 19.7 ± 0.732 |
| **PMB** | **300 IU** | 17.2 ± 0.271 | 23.5 ± 0.437 | 0.000 ± 0.000 | 20.3 ± 0.234 | 20.4 ± 0.557 | 19.7 ± 0.107 | 19.5 ± 0.305 | 17.8 ± 2.69 | 9.66 ± 0.651 | 14.5 ± 0.155 | 14.2 ± 0.338 |
| **VAN** | **30 µg** | 0.000 ± 0.000 | 0.000 ± 0.000 | 0.000 ± 0.000 | 0.000 ± 0.000 | 0.000 ± 0.000 | 0.000 ± 0.000 | 0.000 ± 0.000 | 0.000 ± 0.000 | 24.4 ± 0.945 | 19.2 ± 0.244 | 0.000 ± 0.000 |
| **CLI** | **2 μg** | 0.000 ± 0.000 | 0.000 ± 0.000 | 0.000 ± 0.000 | 0.000 ± 0.000 | 0.000 ± 0.000 | 0.000 ± 0.000 | 0.000 ± 0.000 | 0.000 ± 0.000 | 22.7 ± 1.06 | 19.9 ± 0.302 | 0.000 ± 0.000 |
| **RIF** | **5 µg** | 9.08 ± 0.336 | 13.9 ± 0.211 | 11.6 ± 2.40 | 10.0 ± 0.146 | 10.5 ± 0.270 | 9.18 ± 0.238 | 8.72 ± 0.220 | 9.92 ± 1.82 | 23.1 ± 0.458 | 17.9 ± 0.727 | 9.73 ± 0.378 |
| **CST** | **10 µg** | 14.7 ± 0.111 | 22.2 ± 0.222 | 19.4 ± 0.990 | 18.2 ± 0.228 | 18.7 ± 0.229 | 18.8 ± 0.137 | 18.1 ± 0.211 | 13.6 ± 1.77 | 0.000 ± 0.000 | 0.000 ± 0.000 | 12.9 ± 0.169 |
| **TET (+)** | **200 μg** | 27.8 ± 0.705 | 30.7 ± 0.983 | 29.1 ± 5.37 | 34.0 ± 0.881 | 34.6 ± 1.12 | 28.3 ± 0.489 | 28.6 ± 0.375 | 23.6 ± 3.18 | 36.5 ± 0.789 | 32.8 ± 0.233 | 28.8 ± 0.363 |
| **MeOH (-)** | **20 µL** | 0.000 ± 0.000 | 0.000 ± 0.000 | 0.000 ± 0.000 | 0.000 ± 0.000 | 0.000 ± 0.000 | 0.000 ± 0.000 | 0.000 ± 0.000 | 0.000 ± 0.000 | 0.000 ± 0.000 | 0.000 ± 0.000 | 0.000 ± 0.000 |

*Strains 3 and 8 became RIT 836 and RIT 838, respectively. The data represent the mean values ± SD of two independent experiments, of which one replicate of each is shown in Fig S1.

**Table S2.** Zone of inhibition (ZOI) values measured from disc-diffusion inhibitory assays of *P. rodasii* RIT 836 and *P. endophytica* RIT 838 spent LB medium extracts tested against various bacteria.

| **Conditions/Strain** | | ***P. rodasii*** **RIT 836** | | | | ***P. endophytica*** **RIT 838** | | | |
| --- | --- | --- | --- | --- | --- | --- | --- | --- | --- |
| **Sample** | **Amount** | ***E. coli*** | ***S. aureus*** | ***P. aeruginosa*** | ***B. subtilis*** | ***E. coli*** | ***S. aureus*** | ***P. aeruginosa*** | ***B. subtilis*** |
|  |  | **ZOI (mm)** | | | | **ZOI (mm)** | | | |
| **Crude Extract 1** | **1,250 μg** | 6.35 ± 0.17 | 6.43 ± 0.276 | 6.63 ± 0.156 | 7.12 ± 0.580 | 6.88 ± 0.184 | 6.9 ± 0.516 | 7.69 ± 0.057 | 8.2 ± 0.035 |
| **Crude Extract 2** | **2,500 μg** | 6.72 ± 0.035 | 7.49 ± 0.474 | 8.43 ± 1.336 | 9.11 ± 0.453 | 7.23 ± 0.198 | 9.85 ± 0.778 | 10.3 ± 0.071 | 10.7 ± 0.354 |
| **Crude Extract 3** | **5,000 μg** | 8.2 ± 0.997 | 10.1 ± 0.325 | 10.3 ± 0.594 | 11.6 ± 0.141 | 9.94 ± 0.233 | 12.1 ± 0.778 | 12.7 ± 0.495 | 13.6 ± 0.495 |
| **Tetracycline** | **200 μg** | 17.2 ± 0.919 | 22.7 ± 0.919 | 13.1 ± 0.707 | 20.8 ± 0.495 | 18.6 ± 0.141 | 24 ± 0.424 | 14.4 ± 1.344 | 20.6 ± 0.566 |
| **Blank** | **5,000 μg** | 8.44 ± 0.014 | 9.35 ± 0.085 | 10.2 ± 0.721 | 11.4 ± 0.141 | 8.6 ± 0.113 | 10.1 ± 0.283 | 10.2 ± 0.665 | 11.7 ± 0.424 |
| **Methanol** | **20 μL** | 0 ± 0 | 0 ± 0 | 0 ± 0 | 0 ± 0 | 0 ± 0 | 0 ± 0 | 0 ± 0 | 0 ± 0 |

*The data represent the mean values ± SD of two independent experiments, of which one replicate of each is shown in Fig S2 and S3.

**Table S3.** Summary of all biosynthetic gene cluster regions found in *P. rodasii* RIT 836 and *P. endophytica* RIT 838 antiSMASH analysis.

| **Strain** | **Region** | **Type** | **From** | **To** | **Most Similar Known Cluster** | **Type** | **Similarity** |
| --- | --- | --- | --- | --- | --- | --- | --- |
| RIT 836 | [Region 1.1](https://antismash.secondarymetabolites.org/upload/bacteria-1bc5b7a0-cd42-4d67-a73d-13c58e2074c6/index.html#r1c1) | [redox-cofactor](https://docs.antismash.secondarymetabolites.org/glossary/#redox-cofactor) | 147928 | 170092 | [lankacidin C](https://mibig.secondarymetabolites.org/go/BGC0001100/1) | NRP + Polyketide | 0.13 |
| RIT 836 | [Region 1.2](https://antismash.secondarymetabolites.org/upload/bacteria-1bc5b7a0-cd42-4d67-a73d-13c58e2074c6/index.html#r1c2) | arylpolyene, hserlactone | 474448 | 531049 | [aryl polyenes](https://mibig.secondarymetabolites.org/go/BGC0002008/1) | Other | 0.94 |
| RIT 836 | [Region 1.3](https://antismash.secondarymetabolites.org/upload/bacteria-1bc5b7a0-cd42-4d67-a73d-13c58e2074c6/index.html#r1c3) | thiopeptide,NRPS | 598064 | 670478 | [burkholderic acid](https://mibig.secondarymetabolites.org/go/BGC0001120/1) | NRP + Polyketide:Modular type I | 0.13 |
| RIT 836 | [Region 2.1](https://antismash.secondarymetabolites.org/upload/bacteria-1bc5b7a0-cd42-4d67-a73d-13c58e2074c6/index.html#r2c1) | [NRPS](https://docs.antismash.secondarymetabolites.org/glossary/#nrps) | 706927 | 755332 | - | - | - |
| RIT 836 | [Region 2.2](https://antismash.secondarymetabolites.org/upload/bacteria-1bc5b7a0-cd42-4d67-a73d-13c58e2074c6/index.html#r2c2) | [terpene](https://docs.antismash.secondarymetabolites.org/glossary/#terpene) | 800457 | 824027 | [carotenoid](https://mibig.secondarymetabolites.org/go/BGC0000639/1) | Terpene | 1 |
| RIT 836 | [Region 3.1](https://antismash.secondarymetabolites.org/upload/bacteria-1bc5b7a0-cd42-4d67-a73d-13c58e2074c6/index.html#r3c1) | [NRPS](https://docs.antismash.secondarymetabolites.org/glossary/#nrps) | 325935 | 369846 | [turnerbactin](https://mibig.secondarymetabolites.org/go/BGC0000451/1) | NRP | 0.38 |
| RIT 836 | [Region 8.1](https://antismash.secondarymetabolites.org/upload/bacteria-1bc5b7a0-cd42-4d67-a73d-13c58e2074c6/index.html#r8c1) | [NRPS](https://docs.antismash.secondarymetabolites.org/glossary/#nrps) | 1 | 21634 | - | - | - |
| RIT 836 | [Region 8.2](https://antismash.secondarymetabolites.org/upload/bacteria-1bc5b7a0-cd42-4d67-a73d-13c58e2074c6/index.html#r8c2) | [NRPS](https://docs.antismash.secondarymetabolites.org/glossary/#nrps) | 211952 | 248205 | - | - | - |
| RIT 838 | [Region 5.1](https://antismash.secondarymetabolites.org/upload/bacteria-53499400-a28b-4c5b-852e-a8794541979a/index.html#r5c1) | [thiopeptide](https://docs.antismash.secondarymetabolites.org/glossary/#thiopeptide) | 105835 | 132136 | [O-antigen](https://mibig.secondarymetabolites.org/go/BGC0000781/1) | Saccharide | 0.14 |
| RIT 838 | [Region 8.1](https://antismash.secondarymetabolites.org/upload/bacteria-53499400-a28b-4c5b-852e-a8794541979a/index.html#r8c1) | [arylpolyene](https://docs.antismash.secondarymetabolites.org/glossary/#arylpolyene) | 41506 | 85102 | [APE Ec](https://mibig.secondarymetabolites.org/go/BGC0000836/1) | Other | 0.89 |
| RIT 838 | [Region 11.1](https://antismash.secondarymetabolites.org/upload/bacteria-53499400-a28b-4c5b-852e-a8794541979a/index.html#r11c1) | [NRPS](https://docs.antismash.secondarymetabolites.org/glossary/#nrps) | 135301 | 188248 | [pyoverdin](https://mibig.secondarymetabolites.org/go/BGC0000413/1) | NRP | 0.11 |
| RIT 838 | [Region 11.2](https://antismash.secondarymetabolites.org/upload/bacteria-53499400-a28b-4c5b-852e-a8794541979a/index.html#r11c2) | [T3PKS](https://docs.antismash.secondarymetabolites.org/glossary/#t3pks) | 224215 | 265402 | - | - | - |
| RIT 838 | [Region 12.1](https://antismash.secondarymetabolites.org/upload/bacteria-53499400-a28b-4c5b-852e-a8794541979a/index.html#r12c1) | [RiPP-like](https://docs.antismash.secondarymetabolites.org/glossary/#ripp-like) | 30547 | 41401 | - | - | - |
| RIT 838 | [Region 16.1](https://antismash.secondarymetabolites.org/upload/bacteria-53499400-a28b-4c5b-852e-a8794541979a/index.html#r16c1) | [NRPS](https://docs.antismash.secondarymetabolites.org/glossary/#nrps) | 89945 | 133811 | [qinichelins](https://mibig.secondarymetabolites.org/go/BGC0001752/1) | NRP | 0.11 |
| RIT 838 | [Region 18.1](https://antismash.secondarymetabolites.org/upload/bacteria-53499400-a28b-4c5b-852e-a8794541979a/index.html#r18c1) | [redox-cofactor](https://docs.antismash.secondarymetabolites.org/glossary/#redox-cofactor) | 57562 | 79709 | [lankacidin C](https://mibig.secondarymetabolites.org/go/BGC0001100/1) | NRP + Polyketide | 0.13 |
| RIT 838 | [Region 19.1](https://antismash.secondarymetabolites.org/upload/bacteria-53499400-a28b-4c5b-852e-a8794541979a/index.html#r19c1) | [NAGGN](https://docs.antismash.secondarymetabolites.org/glossary/#naggn) | 118059 | 132792 | - | - | - |
| RIT 838 | [Region 24.1](https://antismash.secondarymetabolites.org/upload/bacteria-53499400-a28b-4c5b-852e-a8794541979a/index.html#r24c1) | NRPS, CDPS | 11117 | 59850 | [pseudomonine](https://mibig.secondarymetabolites.org/go/BGC0000410/1) | NRP | 1 |
| RIT 838 | [Region 29.1](https://antismash.secondarymetabolites.org/upload/bacteria-53499400-a28b-4c5b-852e-a8794541979a/index.html#r29c1) | [NRPS](https://docs.antismash.secondarymetabolites.org/glossary/#nrps) | 58491 | 121988 | [pyoverdin](https://mibig.secondarymetabolites.org/go/BGC0000413/1) | NRP | 0.11 |
| RIT 838 | [Region 47.1](https://antismash.secondarymetabolites.org/upload/bacteria-53499400-a28b-4c5b-852e-a8794541979a/index.html#r47c1) | [NRPS](https://docs.antismash.secondarymetabolites.org/glossary/#nrps) | 12106 | 58447 | [putisolvin](https://mibig.secondarymetabolites.org/go/BGC0000411/1) | NRP | 0.5 |
| RIT 838 | [Region 54.1](https://antismash.secondarymetabolites.org/upload/bacteria-53499400-a28b-4c5b-852e-a8794541979a/index.html#r54c1) | [PpyS-KS](https://docs.antismash.secondarymetabolites.org/glossary/#ppys-ks) | 1 | 14584 | [pseudopyronine A / pseudopyronine B](https://mibig.secondarymetabolites.org/go/BGC0001285/1) | Other:Fatty acid | 0.5 |
| RIT 838 | [Region 57.1](https://antismash.secondarymetabolites.org/upload/bacteria-53499400-a28b-4c5b-852e-a8794541979a/index.html#r57c1) | [ranthipeptide](https://docs.antismash.secondarymetabolites.org/glossary/#ranthipeptide) | 21954 | 43384 | [pyoverdin](https://mibig.secondarymetabolites.org/go/BGC0000413/1) | NRP | 0.07 |
| RIT 838 | [Region 80.1](https://antismash.secondarymetabolites.org/upload/bacteria-53499400-a28b-4c5b-852e-a8794541979a/index.html#r80c1) | NRPS, NRPS-like | 1 | 21907 | [gacamide A](https://mibig.secondarymetabolites.org/go/BGC0001842/1) | NRP:Lipopeptide | 0.85 |
| RIT 838 | [Region 83.1](https://antismash.secondarymetabolites.org/upload/bacteria-53499400-a28b-4c5b-852e-a8794541979a/index.html#r83c1) | [RiPP-like](https://docs.antismash.secondarymetabolites.org/glossary/#ripp-like) | 11922 | 20139 | - | - | - |
| RIT 838 | [Region 88.1](https://antismash.secondarymetabolites.org/upload/bacteria-53499400-a28b-4c5b-852e-a8794541979a/index.html#r88c1) | [NRPS-like](https://docs.antismash.secondarymetabolites.org/glossary/#nrps-like) | 1 | 14971 | [fragin](https://mibig.secondarymetabolites.org/go/BGC0001599/1) | NRP | 0.37 |
| RIT 838 | [Region 92.1](https://antismash.secondarymetabolites.org/upload/bacteria-53499400-a28b-4c5b-852e-a8794541979a/index.html#r92c1) | [NRPS](https://docs.antismash.secondarymetabolites.org/glossary/#nrps) | 1 | 12009 | [rhizomide A / rhizomide B / rhizomide C](https://mibig.secondarymetabolites.org/go/BGC0001758/1) | NRP | 1 |
| RIT 838 | [Region 105.1](https://antismash.secondarymetabolites.org/upload/bacteria-53499400-a28b-4c5b-852e-a8794541979a/index.html#r105c1) | [NRPS](https://docs.antismash.secondarymetabolites.org/glossary/#nrps) | 1 | 5854 | [rhizomide A / rhizomide B / rhizomide C](https://mibig.secondarymetabolites.org/go/BGC0001758/1) | NRP | 1 |
| RIT 838 | [Region 119.1](https://antismash.secondarymetabolites.org/upload/bacteria-53499400-a28b-4c5b-852e-a8794541979a/index.html#r119c1) | [NRPS](https://docs.antismash.secondarymetabolites.org/glossary/#nrps) | 1 | 1827 | [rhizomide A / rhizomide B / rhizomide C](https://mibig.secondarymetabolites.org/go/BGC0001758/1) | NRP | 1 |
| RIT 838 | [Region 120.1](https://antismash.secondarymetabolites.org/upload/bacteria-53499400-a28b-4c5b-852e-a8794541979a/index.html#r120c1) | [NRPS](https://docs.antismash.secondarymetabolites.org/glossary/#nrps) | 1 | 1821 | [rhizomide A / rhizomide B / rhizomide C](https://mibig.secondarymetabolites.org/go/BGC0001758/1) | NRP | 1 |

**Table S4.** MIBiG Comparison, ClusterBlast analysis, and KnownClusterBlast analysis of the genomes of *P. rodasii* RIT 836 and *P. endophytica* RIT 838.

| **Region, Strain** | **Comparison Type** |  |  |  |  |  |  |
| --- | --- | --- | --- | --- | --- | --- | --- |
| **Region 1.2, RIT 836** | **MIBig Comparison** | **Reference** | **Similarity score** | **Type** | **Compound(s)** | **Organism** | **Note** |
|  |  | [BGC0002008.1](https://mibig.secondarymetabolites.org/repository/BGC0002008/index.html#r1c1) | 0.49 | Other | aryl polyenes | *Xenorhabdus doucetiae* |  |
|  | **ClusterBlast** | **antiSMASH Region ID** | **Gene Similarity** | **Type** |  | **Organism** | **Note** |
|  |  | NZ_OBEC01000015 (6534-63199) | 100% | arylpolyene | hserlactone | *Pantoea sp.* GL120224-02 |  |
|  |  | NZ_CP040095 (1557854-1614471) | 100% | arylpolyene | hserlactone | *Pantoea sp.* SO10 |  |
|  |  | NZ_FOSD01000001 (452896-509500) | 100% | arylpolyene | hserlactone | *Pantoea sp.* YR512 |  |
|  |  | NZ_SZZZ01000006 (213292-269915) | 100% | arylpolyene | hserlactone | *Pantoea sp.* Taur |  |
|  |  | NZ_CP011427 (2452217-2508511) | 100% | arylpolyene | hserlactone | *Pantoea vagans* |  |
|  |  | NZ_SLVN01000005 (51741-110552) | 96% | arylpolyene | hserlactone | *Pantoea sp.* BK028 |  |
|  |  | NZ_CP009454 (3173074-3232641) | 95% | arylpolyene | hserlactone | *Pantoea rwandensis* |  |
|  |  | NZ_SCKT01000001 (682267-742066) | 91% | arylpolyene | hserlactone | *Pantoea dispersa* |  |
|  |  | NZ_CP045216 (2791379-2851854) | 90% | arylpolyene | hserlactone | *Pantoea dispersa* |  |
|  |  | NZ_KK403338 (302472-362312) | 83% | arylpolyene | hserlactone | *Pantoea agglomerans* Eh318 |  |
|  | **KnownClusterBlast** | **MIBiG accession** | **Gene Similarity** | **Type** | **Compound** | **Organism** | **Note** |
|  |  | BGC0002008 | 94% | Other | aryl polyenes | *Xenorhabdus doucetiae* |  |
|  |  | BGC0000836 | 84% | Other | APE Ec | *Escherichia coli CFT073* |  |
|  |  |  |  |  |  |  |  |
| **Region 2.2, RIT 836** | **Comparison Type** | **Reference** | **Similarity score** | **Type** | **Compound(s)** | **Organism** | **Note** |
|  | **MIBiG Comparison** | [BGC0000639.1](https://mibig.secondarymetabolites.org/repository/BGC0000639/index.html#r1c1) | 0.79 | terpene | carotenoid | *Enterobacteriaceae bacterium* DC260 |  |
|  | **ClusterBlast** | **antiSMASH Region ID** | **Gene Similarity** | **Type** |  | **Organism** |  |
|  |  | NZ_CEFO01000004 (116832-140403) | 95% | terpene |  | *Enterobacter ludwigii* strain EnVs6 |  |
|  |  | NZ_CP040096 (400902-424473) | 90% | terpene |  | *Pantoea sp.* SO10 |  |
|  |  | NZ_FOSD01000006 (357221-380792) | 80% | terpene |  | *Pantoea sp.* YR512 |  |
|  | **KnownClusterBlast** | **MIBiG accession** | **Gene Similarity** | **Type** | **Compound** | **Organism** | **Note** |
|  |  | BGC0000639 | 100% | terpene | carotenoid | *Enterobacteriaceae bacterium* DC260 |  |
|  |  | BGC0000641 | 100% | terpene | carotenoid | *Enterobacteriaceae bacterium* DC416 |  |
|  |  | BGC0000638 | 100% | terpene | carotenoid | *Pantoea ananatis* |  |
|  |  | BGC0000642 | 85% | terpene | carotenoid | *Enterobacteriaceae bacterium* DC413 |  |
|  |  | BGC0000640 | 83% | terpene | carotenoid | *Enterobacteriaceae bacterium* DC404 |  |
|  |  | BGC0000656 | 100% | terpene | zeaxanthin | *Xanthobacter autotrophicus* Py2 |  |
|  | **Comparison Type** |  |  |  |  |  |  |
| **Region 8.1, RIT 838** | **MIBiG Comparison** | **Reference** | **Similarity score** | **Type** | **Compound(s)** | **Organism** |  |
|  |  | [BGC0002008.1](https://mibig.secondarymetabolites.org/repository/BGC0002008/index.html#r1c1) | 0.5 | Other | aryl polyenes | *Xenorhabdus doucetiae* |  |
|  | **ClusterBlast** | **antiSMASH Region ID** | **Gene Similarity** | **Type** |  | **Organism** | **Note** |
|  |  | NZ_QKME01000004 (10675-54272) | 100% | arylpolyene |  | *Scandinavium goeteborgense* |  |
|  |  | NZ_CP054058 (209317-252914) | 95% | arylpolyene |  | *Scandinavium goeteborgense* |  |
|  |  | NZ_FKGP01000008 (121564-165155) | 84% | arylpolyene |  | *Enterobacter hormaechei* |  |
|  |  | NZ_KI973154 (692843-736434) | 82% | arylpolyene |  | *Enterobacter hormaechei* |  |
|  |  | NZ_CP049046 (3444683-3488274) | 82% | arylpolyene |  | *Enterobacter hormaechei* |  |
|  |  | NZ_LS999206 (4525088-4568679) | 82% | arylpolyene |  | *Enterobacter hormaechei* |  |
|  |  | NZ_JAALLM010000011 (145833-189424) | 82% | arylpolyene |  | *Enterobacter hormaechei* |  |
|  |  | NZ_KI973142 (50693-94284) | 82% | arylpolyene |  | *Enterobacter hormaechei* |  |
|  |  | NZ_ANID01000005 (167063-210654) | 82% | arylpolyene |  | *Enterobacter hormaechei* |  |
|  |  | NZ_CP044107 (830698-874289) | 82% | arylpolyene |  | *Enterobacter hormaechei* |  |
|  | **KnownClusterBlast** | **MIBiG accession** | **Gene Similarity** | **Type** | **Compound** | **Organism** |  |
|  |  | BGC0000836 | 89% | Other | APE Ec | *Escherichia coli* CFT073 |  |
|  |  | BGC0002008 | 88% | Other | aryl polyenes | *Xenorhabdus doucetiae* |  |
|  | **Comparison Type** |  |  |  |  |  |  |
| **Region 24.1, RIT 838** | **MIBiG Comparison** | **Reference** | **Similarity score** | **Type** | **Compound(s)** | **Organism** | **Note** |
|  |  | [BGC0000410.1](https://mibig.secondarymetabolites.org/repository/BGC0000410/index.html#r1c1) | 0.58 | NRP | pseudomonine | *Pseudomonas fluorescens* |  |
|  | **ClusterBlast** | **antiSMASH Region ID** | **Gene Similarity** | **Type** |  | **Organism** | **Note** |
|  |  | NZ_QKZD01000009 (181329-232204) | 94% | CDPS, NRPS |  | *Pseudomonas sp.* 2848 |  |
|  |  | NZ_CP023299 (5587690-5638712) | 89% | CDPS, NRPS |  | *Pseudomonas mosselii* |  |
|  |  | NZ_LN847264 (2983947-3034262) | 89% | CDPS, NRPS |  | *Pseudomonas sp.* CCOS 191 |  |
|  |  | NZ_QJRO01000019 (37756-88631) | 87% | CDPS, NRPS |  | *Pseudomonas soli* |  |
|  | **KnownClusterBlast** | **MIBiG accession** | **Gene Similarity** | **Type** | **Compound** | **Organism** | **Note** |
|  |  | BGC0000410 | 100% | NRP | pseudomonine | *Pseudomonas fluorescens* |  |
|  | **Comparison Type** |  |  |  |  |  |  |
| **Region 80.1, RIT 838** | **MIBiG Comparison** | **Reference** | **Similarity score** | **Type** | **Compound(s)** | **Organism** | **Note** |
|  |  | [BGC0000463.1](https://mibig.secondarymetabolites.org/repository/BGC0000463/index.html#r1c1) | 1.04 | NRP | xantholysin A, xantholysin B, xantholysin C | *Pseudomonas putida* | Reasons for retirement: Entry is spread over multiple contigs |
|  |  | [BGC0000389.1](https://mibig.secondarymetabolites.org/repository/BGC0000389/index.html#r1c1) | 0.82 | NRP | massetolide A | *Pseudomonas fluorescens* SS101 | Reasons for retirement: Entry is spread over multiple contigs |
|  |  | [BGC0001833.1](https://mibig.secondarymetabolites.org/repository/BGC0001833/index.html#r1c1) | 0.80 | NRP | icosalide A, icosalide B | *Burkholderia gladioli* |  |
|  | **ClusterBlast** | **antiSMASH Region ID** | **Gene Similarity** | **Type** |  | **Organism** | **Note** |
|  |  | NZ_CP019952 (2429401-2508266) | 29% | NRPS |  | *Pseudomonas parafulva* |  |
|  |  | NZ_SMCF01000010 (43468-123610) | 29% | NRPS |  | *Pseudomonas sp.* LP_8_YM |  |
|  |  | NZ_QJRU01000004 (162256-242398) | 29% | NRPS |  | *Pseudomonas fulva* |  |
|  |  | NZ_CP014025 (2233320-2312105) | 29% | NRPS |  | *Pseudomonas fulva* |  |
|  |  | NZ_CP025035 (2248813-2327279) | 27% | NRPS |  | Pseudomonas sp. *SGAir0191* |  |
|  |  | NZ_KE384443 (360137-440296) | 26% | NRPS |  | *Pseudomonas parafulva* NBRC 16636 = DSM 17004 |  |
|  | **KnownClusterBlast** | **MIBiG accession** | **Gene Similarity** | **Type** | **Compound** | **Organism** | **Note** |
|  |  | BGC0000435 | 100% | NRP | syringafactin | *Pseudomonas syringae pv. tomato str.* DC3000 |  |
|  |  | BGC0001842 | 85% | NRP:Lipopeptide | gacamide A | *Pseudomonas fluorescens* Pf0-1 |  |
|  |  | BGC0000323 | 83% | NRP:Lipopeptide | cichofactin A / cichofactin B | *Pseudomonas cichorii* |  |
|  | **Comparison Type** |  |  |  |  |  |  |
| **Region 92.1, RIT838** | **MIBiG Comparison** | **Reference** | **Similarity score** | **Type** | **Compound(s)** | **Organism** | **Note** |
|  |  | [BGC0000457.1](https://mibig.secondarymetabolites.org/repository/BGC0000457/index.html#r1c1) | 0.27 | NRP | vicibactin | Rhizobium etli *CFN 42* |  |
|  |  | [BGC0001128.1](https://mibig.secondarymetabolites.org/repository/BGC0001128/index.html#r1c1) | 0.27 | NRP | luminmide | *Photorhabdus laumondii* subsp. laumondii TTO1 | Luminmide also known as gamexpeptide C |
|  | **ClusterBlast** | **antiSMASH Region ID** | **Gene Similarity** | **Type** |  | **Organism** | **Note** |
|  |  | NZ_QJRO01000010 (82317-162294) | 23% | NRPS |  | *Pseudomonas soli* |  |
|  |  | NZ_LN847264 (2657716-2737085) | 23% | NRPS |  | *Pseudomonas sp.* CCOS 191 |  |
|  |  | NZ_CP026386 (613094-691966) | 22% | NRPS |  | *Pseudomonas sp.* PONIH3 |  |
|  |  | NZ_CP009365 (1723810-1802704) | 21% | NRPS |  | *Pseudomonas soli* |  |
|  | **KnownClusterBlast** | **MIBiG accession** | **Gene Similarity** | **Type** | **Compound** | **Organism** | **Note** |
|  |  | BGC0001758 | 100% | NRP | rhizomide A / rhizomide B / rhizomide C | *Paraburkholderia rhizoxinica* HKI 454 |  |
|  |  | BGC0001128 | 100% | NRP | luminmide | *Photorhabdus laumondii* subsp. laumondii TTO1 |  |
|  |  | BGC0001135 | 100% | NRP | bicornutin A1 / bicornutin A2 | *Xenorhabdus budapestensis* |  |

**Table S5.** Summary of all biosynthetic gene cluster regions found in *P. rodasii* ND03, *P. rodasii* LMG 26273, *P. rodasii* DSM 26611, and *P. endophytica* BSTT44 antiSMASH analysis.

| **Strain** | **Region** | **Type** | **From** | **To** | **Most Similar Known Cluster** | **Type** | **Similarity** |
| --- | --- | --- | --- | --- | --- | --- | --- |
| *Pantoea rodasii* ND03 | [Region 5.1](https://antismash.secondarymetabolites.org/upload/bacteria-f06bfdf4-e97a-458d-99a4-0d30eee7f4da/index.html#r5c1) | NRP-metallophore,NRPS | 25,141 | 78,845 | [trichrysobactin/cyclic trichrysobactin/chrysobactin/dichrysobactin](https://mibig.secondarymetabolites.org/go/BGC0002414/1) | NRP | 84% |
| *Pantoea rodasii* ND03 | [Region 17.1](https://antismash.secondarymetabolites.org/upload/bacteria-f06bfdf4-e97a-458d-99a4-0d30eee7f4da/index.html#r17c1) | [hserlactone](https://docs.antismash.secondarymetabolites.org/glossary/#hserlactone) | 31,211 | 51,843 |  |  |  |
| *Pantoea rodasii* ND03 | [Region 21.1](https://antismash.secondarymetabolites.org/upload/bacteria-f06bfdf4-e97a-458d-99a4-0d30eee7f4da/index.html#r21c1) | arylpolyene,hserlactone | 1 | 42,116 | [aryl polyenes](https://mibig.secondarymetabolites.org/go/BGC0002008/1) | Other | 88% |
| *Pantoea rodasii* ND03 | [Region 38.1](https://antismash.secondarymetabolites.org/upload/bacteria-f06bfdf4-e97a-458d-99a4-0d30eee7f4da/index.html#r38c1) | [NRPS](https://docs.antismash.secondarymetabolites.org/glossary/#nrps) | 43,070 | 70,766 | [trichrysobactin/cyclic trichrysobactin/chrysobactin/dichrysobactin](https://mibig.secondarymetabolites.org/go/BGC0002414/1) | NRP | 30% |
| *Pantoea rodasii* ND03 | [Region 51.1](https://antismash.secondarymetabolites.org/upload/bacteria-f06bfdf4-e97a-458d-99a4-0d30eee7f4da/index.html#r51c1) | [thiopeptide](https://docs.antismash.secondarymetabolites.org/glossary/#thiopeptide) | 11,184 | 37,395 | [O-antigen](https://mibig.secondarymetabolites.org/go/BGC0000781/1) | Saccharide | 14% |
| *Pantoea rodasii* ND03 | [Region 88.1](https://antismash.secondarymetabolites.org/upload/bacteria-f06bfdf4-e97a-458d-99a4-0d30eee7f4da/index.html#r88c1) | NRP-metallophore,NRPS | 1 | 46,948 | [enantio-pyochelin](https://mibig.secondarymetabolites.org/go/BGC0002475/1) | NRP | 30% |
| *Pantoea rodasii* ND03 | [Region 95.1](https://antismash.secondarymetabolites.org/upload/bacteria-f06bfdf4-e97a-458d-99a4-0d30eee7f4da/index.html#r95c1) | [terpene](https://docs.antismash.secondarymetabolites.org/glossary/#terpene) | 6,699 | 24,239 | [carotenoid](https://mibig.secondarymetabolites.org/go/BGC0000639/1) | Terpene | 100% |
| *Pantoea rodasii* ND03 | [Region 102.1](https://antismash.secondarymetabolites.org/upload/bacteria-f06bfdf4-e97a-458d-99a4-0d30eee7f4da/index.html#r102c1) | [redox-cofactor](https://docs.antismash.secondarymetabolites.org/glossary/#redox-cofactor) | 3,027 | 19,042 | [lankacidin C](https://mibig.secondarymetabolites.org/go/BGC0001100/1) | NRP+Polyketide | 13% |
| *Pantoea rodasii* LMG 26273 | [Region 4.1](https://antismash.secondarymetabolites.org/upload/bacteria-731a1ef3-f464-4c52-9703-959eb988ff0c/index.html#r4c1) | [NRPS](https://docs.antismash.secondarymetabolites.org/glossary/#nrps) | 249,542 | 293,456 | [trichrysobactin/cyclic trichrysobactin/chrysobactin/dichrysobactin](https://mibig.secondarymetabolites.org/go/BGC0002414/1) | NRP | 76% |
| *Pantoea rodasii* LMG 26273 | [Region 5.1](https://antismash.secondarymetabolites.org/upload/bacteria-731a1ef3-f464-4c52-9703-959eb988ff0c/index.html#r5c1) | [thiopeptide](https://docs.antismash.secondarymetabolites.org/glossary/#thiopeptide) | 75,449 | 101,671 | [O-antigen](https://mibig.secondarymetabolites.org/go/BGC0000781/1) | Saccharide | 14% |
| *Pantoea rodasii* LMG 26273 | [Region 5.2](https://antismash.secondarymetabolites.org/upload/bacteria-731a1ef3-f464-4c52-9703-959eb988ff0c/index.html#r5c2) | [hserlactone](https://docs.antismash.secondarymetabolites.org/glossary/#hserlactone) | 168,389 | 189,000 |  |  |  |
| *Pantoea rodasii* LMG 26273 | [Region 5.3](https://antismash.secondarymetabolites.org/upload/bacteria-731a1ef3-f464-4c52-9703-959eb988ff0c/index.html#r5c3) | [arylpolyene](https://docs.antismash.secondarymetabolites.org/glossary/#arylpolyene) | 223,043 | 266,624 | [aryl polyenes](https://mibig.secondarymetabolites.org/go/BGC0002008/1) | Other | 88% |
| *Pantoea rodasii* LMG 26273 | [Region 6.1](https://antismash.secondarymetabolites.org/upload/bacteria-731a1ef3-f464-4c52-9703-959eb988ff0c/index.html#r6c1) | [RRE-containing](https://docs.antismash.secondarymetabolites.org/glossary/#rre-containing) | 94,420 | 114,698 | [lankacidin C](https://mibig.secondarymetabolites.org/go/BGC0001100/1) | NRP+Polyketide | 13% |
| *Pantoea rodasii* LMG 26273 | [Region 8.1](https://antismash.secondarymetabolites.org/upload/bacteria-731a1ef3-f464-4c52-9703-959eb988ff0c/index.html#r8c1) | [NI-siderophore](https://docs.antismash.secondarymetabolites.org/glossary/#ni-siderophore) | 104,926 | 119,339 | [aerobactin](https://mibig.secondarymetabolites.org/go/BGC0001498/1) | Other | 66% |
| *Pantoea rodasii* LMG 26273 | [Region 9.1](https://antismash.secondarymetabolites.org/upload/bacteria-731a1ef3-f464-4c52-9703-959eb988ff0c/index.html#r9c1) | [NRPS](https://docs.antismash.secondarymetabolites.org/glossary/#nrps) | 82,180 | 130,570 | [trichrysobactin/cyclic trichrysobactin/chrysobactin/dichrysobactin](https://mibig.secondarymetabolites.org/go/BGC0002414/1) | NRP | 38% |
| *Pantoea rodasii* LMG 26273 | [Region 5.3](https://antismash.secondarymetabolites.org/upload/bacteria-731a1ef3-f464-4c52-9703-959eb988ff0c/index.html#r5c3) | [arylpolyene](https://docs.antismash.secondarymetabolites.org/glossary/#arylpolyene) | 223,043 | 266,624 | [aryl polyenes](https://mibig.secondarymetabolites.org/go/BGC0002008/1) | Other | 88% |
| *Pantoea rodasii* LMG 26273 | [Region 15.1](https://antismash.secondarymetabolites.org/upload/bacteria-731a1ef3-f464-4c52-9703-959eb988ff0c/index.html#r15c1) | [RiPP-like](https://docs.antismash.secondarymetabolites.org/glossary/#ripp-like) | 1 | 8,158 |  |  |  |
| *Pantoea rodasii* LMG 26273 | [Region 20.1](https://antismash.secondarymetabolites.org/upload/bacteria-731a1ef3-f464-4c52-9703-959eb988ff0c/index.html#r20c1) | [terpene](https://docs.antismash.secondarymetabolites.org/glossary/#terpene) | 35,534 | 59,107 | [carotenoid](https://mibig.secondarymetabolites.org/go/BGC0000639/1) | Terpene | 100% |
| *Pantoea rodasii* LMG 26273 | [Region 25.1](https://antismash.secondarymetabolites.org/upload/bacteria-731a1ef3-f464-4c52-9703-959eb988ff0c/index.html#r25c1) | [hserlactone](https://docs.antismash.secondarymetabolites.org/glossary/#hserlactone),[cyanobactin](https://docs.antismash.secondarymetabolites.org/glossary/#cyanobactin) | 20,561 | 33,542 |  |  |  |
| *Pantoea rodasii* DSM 26611 | [Region 1.1](https://antismash.secondarymetabolites.org/upload/bacteria-85577ff2-e349-4623-bc6f-b77b5385e50b/index.html#r1c1) | [terpene](https://docs.antismash.secondarymetabolites.org/glossary/#terpene) | 35,661 | 59,234 | [carotenoid](https://mibig.secondarymetabolites.org/go/BGC0000639/1) | Terpene | 100% |
| *Pantoea rodasii* DSM 26611 | [Region 3.1](https://antismash.secondarymetabolites.org/upload/bacteria-85577ff2-e349-4623-bc6f-b77b5385e50b/index.html#r3c1) | [redox-cofactor](https://docs.antismash.secondarymetabolites.org/glossary/#redox-cofactor) | 233,034 | 255,198 | [lankacidin C](https://mibig.secondarymetabolites.org/go/BGC0001100/1) | NRP+Polyketide | 13% |
| *Pantoea rodasii* DSM 26611 | [Region 13.1](https://antismash.secondarymetabolites.org/upload/bacteria-85577ff2-e349-4623-bc6f-b77b5385e50b/index.html#r13c1) | [hserlactone](https://docs.antismash.secondarymetabolites.org/glossary/#hserlactone) | 13,829 | 27,945 |  |  |  |
| *Pantoea rodasii* DSM 26611 | [Region 18.1](https://antismash.secondarymetabolites.org/upload/bacteria-85577ff2-e349-4623-bc6f-b77b5385e50b/index.html#r18c1) | [RiPP-like](https://docs.antismash.secondarymetabolites.org/glossary/#ripp-like) | 108,690 | 117,020 |  |  |  |
| *Pantoea rodasii* DSM 26611 | [Region 23.1](https://antismash.secondarymetabolites.org/upload/bacteria-85577ff2-e349-4623-bc6f-b77b5385e50b/index.html#r23c1) | [thiopeptide](https://docs.antismash.secondarymetabolites.org/glossary/#thiopeptide) | 530,449 | 556,673 | [O-antigen](https://mibig.secondarymetabolites.org/go/BGC0000781/1) | Saccharide | 14% |
| *Pantoea rodasii* DSM 26611 | [Region 23.2](https://antismash.secondarymetabolites.org/upload/bacteria-85577ff2-e349-4623-bc6f-b77b5385e50b/index.html#r23c2) | [hserlactone](https://docs.antismash.secondarymetabolites.org/glossary/#hserlactone) | 623,409 | 644,020 |  |  |  |
| *Pantoea rodasii* DSM 26611 | [Region 24.1](https://antismash.secondarymetabolites.org/upload/bacteria-85577ff2-e349-4623-bc6f-b77b5385e50b/index.html#r24c1) | [arylpolyene](https://docs.antismash.secondarymetabolites.org/glossary/#arylpolyene) | 1 | 41,446 | [aryl polyenes](https://mibig.secondarymetabolites.org/go/BGC0002008/1) | Other | 88% |
| *Pantoea rodasii* DSM 26611 | [Region 25.1](https://antismash.secondarymetabolites.org/upload/bacteria-85577ff2-e349-4623-bc6f-b77b5385e50b/index.html#r25c1) | [NI-siderophore](https://docs.antismash.secondarymetabolites.org/glossary/#ni-siderophore) | 1 | 13,116 | [aerobactin](https://mibig.secondarymetabolites.org/go/BGC0001498/1) | Other | 66% |
| *Pantoea rodasii* DSM 26611 | [Region 26.1](https://antismash.secondarymetabolites.org/upload/bacteria-85577ff2-e349-4623-bc6f-b77b5385e50b/index.html#r26c1) | [NRPS](https://docs.antismash.secondarymetabolites.org/glossary/#nrps) | 48,167 | 96,551 | [trichrysobactin/cyclic trichrysobactin/chrysobactin/dichrysobactin](https://mibig.secondarymetabolites.org/go/BGC0002414/1) | NRP | 46% |
| *Pantoea rodasii* DSM 26611 | [Region 31.1](https://antismash.secondarymetabolites.org/upload/bacteria-85577ff2-e349-4623-bc6f-b77b5385e50b/index.html#r31c1) | [NRP-metallophore](https://docs.antismash.secondarymetabolites.org/glossary/#nrp-metallophore),[NRPS](https://docs.antismash.secondarymetabolites.org/glossary/#nrps) | 240,022 | 293,872 | [frederiksenibactin](https://mibig.secondarymetabolites.org/go/BGC0002413/1) | NRP | 84% |
| *Pseudomonas endophytica* BSTT44 | [Region 112.1](https://antismash.secondarymetabolites.org/upload/bacteria-ca968b29-0c2d-4f4d-90a2-c7ca851344fb/index.html#r112c1) | [betalactone](https://docs.antismash.secondarymetabolites.org/glossary/#betalactone) | 98,852 | 122,069 | [fengycin](https://mibig.secondarymetabolites.org/go/BGC0001095/1) | NRP | 20% |
| *Pseudomonas endophytica* BSTT44 | [Region 135.1](https://antismash.secondarymetabolites.org/upload/bacteria-ca968b29-0c2d-4f4d-90a2-c7ca851344fb/index.html#r135c1) | [transAT-PKS-like](https://docs.antismash.secondarymetabolites.org/glossary/#transat-pks-like) | 15,667 | 47,567 | [Region 135.1](https://antismash.secondarymetabolites.org/upload/bacteria-ca968b29-0c2d-4f4d-90a2-c7ca851344fb/index.html#r135c1) | [transAT-PKS-like](https://docs.antismash.secondarymetabolites.org/glossary/#transat-pks-like) | 15,667 |
| *Pseudomonas endophytica* BSTT44 | [Region 151.1](https://antismash.secondarymetabolites.org/upload/bacteria-ca968b29-0c2d-4f4d-90a2-c7ca851344fb/index.html#r151c1) | [RiPP-like](https://docs.antismash.secondarymetabolites.org/glossary/#ripp-like) | 6,647 | 18,857 | [Region 151.1](https://antismash.secondarymetabolites.org/upload/bacteria-ca968b29-0c2d-4f4d-90a2-c7ca851344fb/index.html#r151c1) | [RiPP-like](https://docs.antismash.secondarymetabolites.org/glossary/#ripp-like) | 6,647 |
| *Pseudomonas endophytica* BSTT44 | [Region 178.1](https://antismash.secondarymetabolites.org/upload/bacteria-ca968b29-0c2d-4f4d-90a2-c7ca851344fb/index.html#r178c1) | [NAGGN](https://docs.antismash.secondarymetabolites.org/glossary/#naggn) | 19,701 | 29,226 | [Region 178.1](https://antismash.secondarymetabolites.org/upload/bacteria-ca968b29-0c2d-4f4d-90a2-c7ca851344fb/index.html#r178c1) | [NAGGN](https://docs.antismash.secondarymetabolites.org/glossary/#naggn) | 19,701 |
| *Pseudomonas endophytica* BSTT44 | [Region 187.1](https://antismash.secondarymetabolites.org/upload/bacteria-ca968b29-0c2d-4f4d-90a2-c7ca851344fb/index.html#r187c1) | [NI-siderophore](https://docs.antismash.secondarymetabolites.org/glossary/#ni-siderophore) | 1 | 8,651 | [Region 187.1](https://antismash.secondarymetabolites.org/upload/bacteria-ca968b29-0c2d-4f4d-90a2-c7ca851344fb/index.html#r187c1) | [NI-siderophore](https://docs.antismash.secondarymetabolites.org/glossary/#ni-siderophore) | 1 |
| *Pseudomonas endophytica* BSTT44 | [Region 218.1](https://antismash.secondarymetabolites.org/upload/bacteria-ca968b29-0c2d-4f4d-90a2-c7ca851344fb/index.html#r218c1) | [redox-cofactor](https://docs.antismash.secondarymetabolites.org/glossary/#redox-cofactor) | 1 | 18,129 | [Region 218.1](https://antismash.secondarymetabolites.org/upload/bacteria-ca968b29-0c2d-4f4d-90a2-c7ca851344fb/index.html#r218c1) | [redox-cofactor](https://docs.antismash.secondarymetabolites.org/glossary/#redox-cofactor) | 1 |
